# Alterations in cardiac DNA methylation in human dilated cardiomyopathy

**DOI:** 10.1002/emmm.201201553

**Published:** 2013-01-22

**Authors:** Jan Haas, Karen S Frese, Yoon Jung Park, Andreas Keller, Britta Vogel, Anders M Lindroth, Dieter Weichenhan, Jennifer Franke, Simon Fischer, Andrea Bauer, Sabine Marquart, Farbod Sedaghat-Hamedani, Elham Kayvanpour, Doreen Köhler, Nadine M Wolf, Sarah Hassel, Rouven Nietsch, Thomas Wieland, Philipp Ehlermann, Jobst-Hendrik Schultz, Andreas Dösch, Derliz Mereles, Stefan Hardt, Johannes Backs, Jörg D Hoheisel, Christoph Plass, Hugo A Katus, Benjamin Meder

**Affiliations:** 1 Department of Internal Medicine III, University of HeidelbergHeidelberg, Germany; 2 Division of Epigenomics and Cancer Risk Factors, German Cancer Research Center (DKFZ)Heidelberg, Germany; 3 Department of Nutritional Science and Food Management, Ewha Womans UniversitySeoul, South Korea; 4 Department of Human Genetics, Saarland UniversityGermany; 5 Division of Functional Genome Analysis, German Cancer Research Center (DKFZ)Heidelberg, Germany; 6 Medical Faculty Mannheim, Institute of Experimental and Clinical Pharmacology and Toxicology, Heidelberg UniversityMannheim, Germany; 7 Department of General Internal Medicine and Psychosomatics, University Hospital HeidelbergHeidelberg, Germany; 8 DZHK (German Centre for Cardiovascular Research), partner site Heidelberg/MannheimMannheim, Germany; 9 DZHK (German Centre for Cardiovascular Research), partner site Heidelberg/MannheimHeidelberg, Germany

**Keywords:** biomarker, dilated cardiomyopathy, DNA methylation, epigenetics, heart failure

## Abstract

Dilated cardiomyopathies (DCM) show remarkable variability in their age of onset, phenotypic presentation, and clinical course. Hence, disease mechanisms must exist that modify the occurrence and progression of DCM, either by genetic or epigenetic factors that may interact with environmental stimuli. In the present study, we examined genome-wide cardiac DNA methylation in patients with idiopathic DCM and controls. We detected methylation differences in pathways related to heart disease, but also in genes with yet unknown function in DCM or heart failure, namely *Lymphocyte antigen 75* (*LY75*), *Tyrosine kinase-type cell surface receptor HER3* (*ERBB3*), *Homeobox B13* (*HOXB13*) and *Adenosine receptor A2A* (*ADORA2A*). Mass-spectrometric analysis and bisulphite-sequencing enabled confirmation of the observed DNA methylation changes in independent cohorts. Aberrant DNA methylation in DCM patients was associated with significant changes in *LY75* and *ADORA2A* mRNA expression, but not in *ERBB3* and *HOXB13*. *In vivo* studies of orthologous *ly75* and *adora2a* in zebrafish demonstrate a functional role of these genes in adaptive or maladaptive pathways in heart failure.

## INTRODUCTION

Idiopathic dilated cardiomyopathy (DCM) is a frequent heart muscle disease with an estimated prevalence of 1:2500 (Karkkainen & Peuhkurinen, 2007). The progressive nature of the disorder is responsible for nearly 50,000 hospitalizations and 10,000 deaths per year in the US alone and is the main cause for heart transplantation in young adults (Dec & Fuster, 1994). Overall, the incidence of the disease has continually increased over the past years and it was recognized that DCM has a substantial genetic contribution (Grunig et al, 1998). It is estimated that about 30–40% of all DCM cases show familial aggregation and until now more than 40 different genes were found to cause genetic DCM (Meder & Katus, 2012). However, since the course of even monogenetic DCM is highly variable, genetic modifiers are thought to have an important influence on phenotypic characteristics and outcome (Friedrichs et al, 2009; Villard et al, 2011). Accordingly, several studies have now identified common genetic polymorphisms that are associated with DCM or heart failure (Friedrichs et al, 2009; Villard et al, 2011).

Disease modification through epigenetic alterations has been convincingly demonstrated for a number of diseases (Feinberg & Tycko, 2004; Jones & Baylin, 2002). In the cardiovascular system, histone modifications and chromatin remodelling are thought to direct adaptive as well as maladaptive molecular pathways in cardiac hypertrophy and failure (Montgomery et al, 2007), and DNA methylation was found to be responsible for the hypermutability of distinct cardiac genes (Meurs & Kuan, 2011). Furthermore, recent studies have highlighted potential interplay between environmental factors and the disease phenotype by epigenetic mechanisms (Herceg & Vaissiere, 2011; Jirtle & Skinner, 2007). However, the knowledge about the impact of epigenetic alterations on the disease phenotype in human patients is still very limited.

The present study investigated for the first time the impact of genome-wide cardiac DNA methylation on human DCM in patients. We identified several candidate genes with altered methylation status and replicated these findings in an independent cohort of DCM patients and controls. Using gene expression analysis and zebrafish as an *in vivo* model, we could furthermore show that appropriate mRNA levels of *LY75* and *ADORA2A* are important for unconstrained cardiac function.

## RESULTS

### DNA methylation is altered in patients with DCM

**Figure 1:**
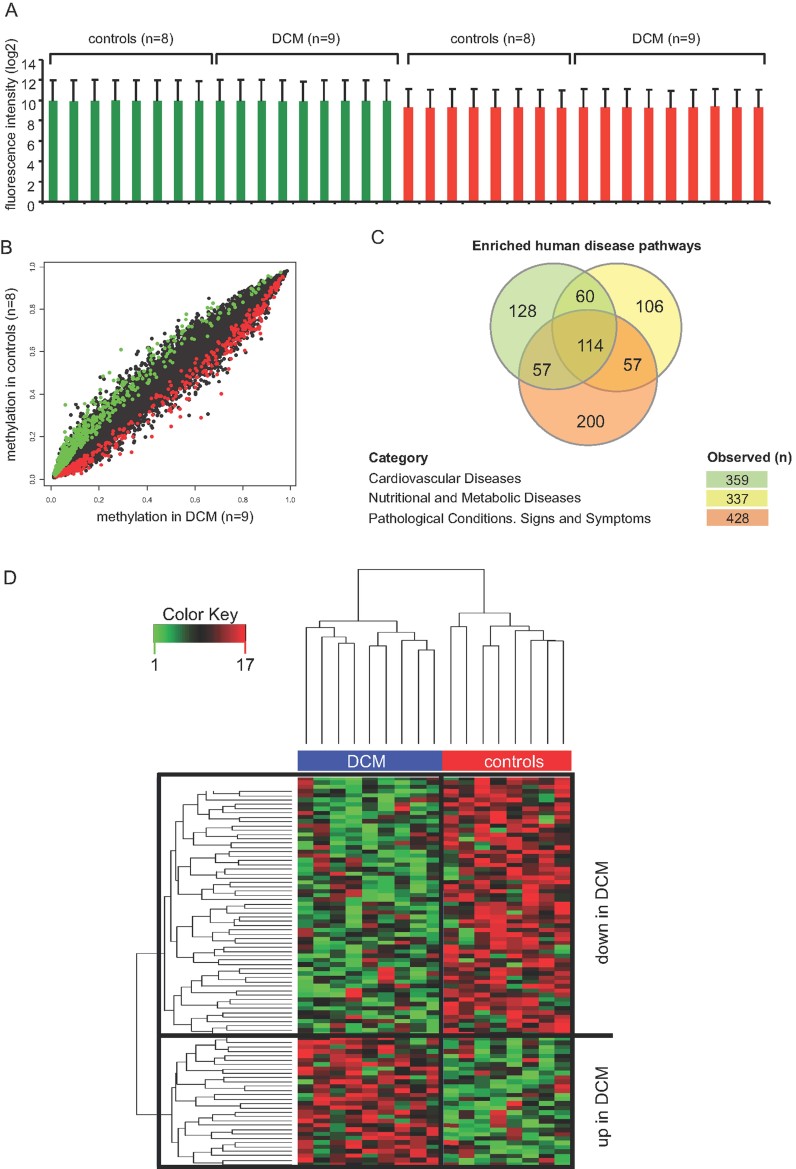
Detection of DNA methylation patterns Bar graphs showing quantile normalized bead colour signals (green and red) from methylation measurements from all patients and controls of the screening stage included in the further analysis. Signal quality was highly similar over all DCM and control samples. Error bars indicate standard deviation.Correlation plot showing the percentage of CpG methylation in controls *versus* DCM patients, resulting in an overall very high correlation. The coloured signals that are furthest away from the bisecting line show significantly hyper- (red) and hypo-methylated (green) CpGs in DCM patients.Gene-set enrichment analysis for NIA human disease pathways. The area-proportional Venn diagram shows that methylation changes in cardiovascular disease genes are significantly enriched together with the overlap of the other indicated gene sets.Cluster analysis for genes with known expression in the human heart and significantly altered methylation. The colour code used for the heatmap is shown in the upper left corner, values range from 1 (sample with the lowest methylation for the considered genes) to 17 (sample with the highest methylation for the considered genes).

We performed two-staged, funnel-like DNA methylation mapping in non-ischaemic, idiopathic DCM patients and controls (Table 1). In the screening stage, we assessed genome-wide DNA methylation levels of CpG islands (CGIs) using the Infinium HumanMethylation 27 platform. We first extracted 1000 ng of genomic DNA from LV biopsies from 10 DCM patients and 10 controls. After methylation profiling, 17 datasets passed the stringent quality filter criteria, exemplarily shown by reaching highly similar bead color signal intensities (Fig 1A). Fig 1B shows a correlation plot of the 27,578 individual methylation sites for all further analysed patients and controls. While the degree of methylation for most CpG sites is highly correlated between the two groups, we detected several CGIs that are hypo- (green dots) or hyper-methylated (red dots) in DCM compared to the controls (unadjusted *p*-value <0.05).

**Table 1:** Study sample characteristics of the screening and replication cohorts

Cohort	*N*	Female (%)	Age (years)	LV-EF (%)
Screening				
DCM cases	9	33	57 ± 5.4	27 ± 7.5
Controls	8	38	42 ± 14	60 ± 4.0
Replication				
DCM cases	30	37	58 ± 14	25.6 ± 8.4
Controls	28	46	57 ± 12	61.5 ± 5.3

For the control patients: Female, female donor status of transplanted heart; LV-EF, left ventricular ejection fraction.

We used the comprehensive screening datasets in a gene set enrichment analysis (GSEA) and identified within the top three (ranked by *p* values) enriched disease categories provided by the National Institute of Aging (NIA) the disease pathways ‘cardiovascular disease’, ‘metabolic disease’ and ‘pathological conditions’, all associated with DCM and with considerable overlaps between each other (Fig 1C). To detect patterns of genes with differential methylation in the screening cohorts, we carried out a clustering approach on genes with known abundant expression in the human heart (http://c-it.mpi-bn.mpg.de). From the annotated 2018 individual genes, 1858 (92.1%) were covered by the applied Infinium assay. Since the degrees of methylation for the genes were not normally distributed, we used two-tailed Wilcoxon rank-sum test to compute a significance value for each gene, which resulted in a total of 90 genes surpassing statistical significance (*p*-value <0.05). While about one-third showed increased methylation in DCM patients, approximately two-thirds were significantly less methylated. Fig 1D gives a graphical representation of differential methylation patterns by using hierarchical clustering on the Euclidian distance. The heat map shows the patterns of higher- and lower methylated genes in DCM patients and controls.

**Figure 2:**
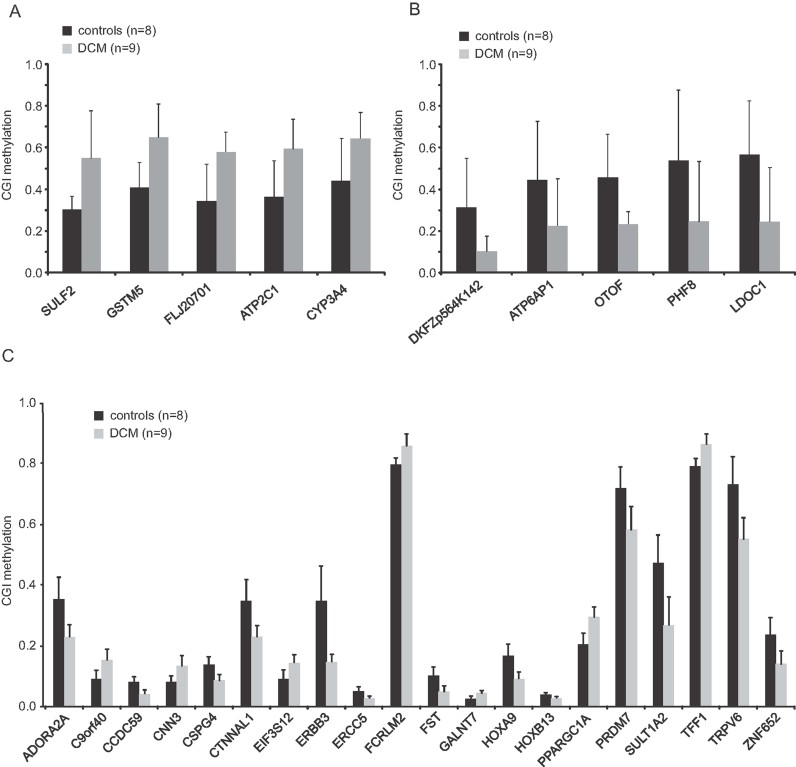
Differentially methylated genes in DCM patients **A, B.** Bar graphs showing the degree of methylation of CGIs of the screening cohort (*n* = 9 DCM patients; *n* = 8 controls). The five genes with the largest increase in methylation in DCM patients are shown in (A) and genes with the largest decrease in methylation are displayed in (B).**C.** The 20 genes with the most significant methylation changes in the screening phase. Error bars indicate standard deviation.

For the replication stage in an independent cohort, methylation patterns of single genes were used to define candidates for a mass spectrometry-based fine-mapping. Fig 2A and B shows the five most hyper- and hypomethylated genes, while Fig 2C details the 20 most significantly dys-methylated genes of the screening stage. The final selection was based on unadjusted *p* values or absolute methylation difference, CGI localization, capability to design specific assay probes, and known expression in the heart.

### Validation of aberrant DNA methylation in DCM

**Figure 3:**
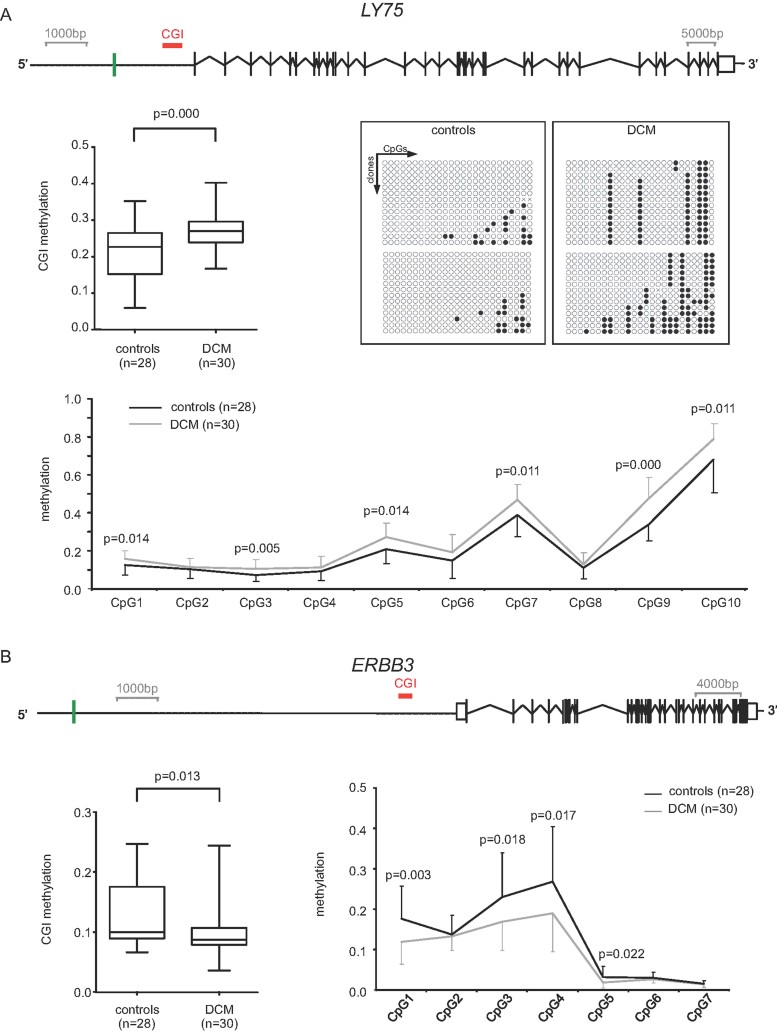
MassARRAY-based validation of differential methylation in *LY75* and *ERBB3* DCM patients show significantly increased DNA methylation in *LY75* (A), while *ERBB3* is significantly lower methylated (B). The schemas above the methylation graphs represent the tested CGI (red lines), in relation to the predicted transcription start-site (green lines), the exons (black bars) and alternatively spliced exons (white boxes) of the genes. The box and whiskers plot (min to max) on the upper left side represents the mean CpG methylation measured by MassARRAY, the individual CpG methylation is shown at the bottom. The pattern of CpG methylation in multiple clones is shown on the upper right insert.The box and whiskers plot (min to max) on the left side represents the mean CpG methylation measured by MassARRAY, the individual CpG methylation is shown on the right.

**Figure 4:**
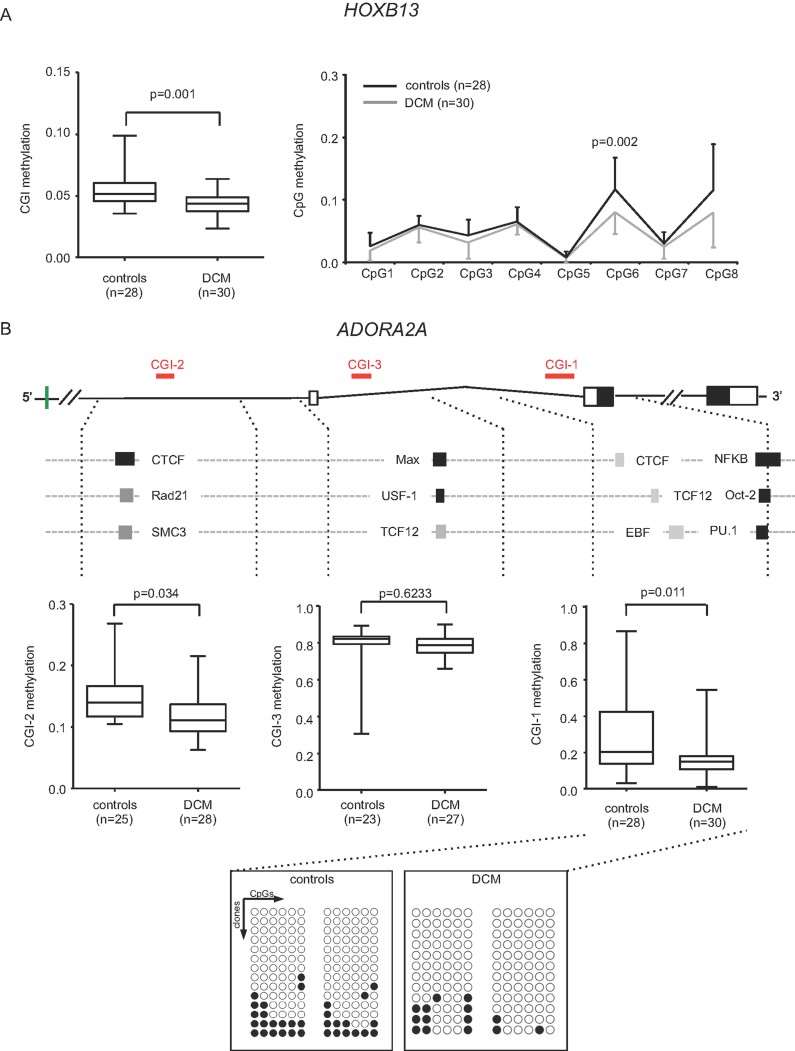
MassARRAY-based validation of differential methylation in *HOXB13* and *ADORA2A* DCM patients show significantly decreased DNA methylation in *HOXB13* (A) and *ADORA2A* (B). The box and whiskers plot (min to max) on the left side represents the mean CpG methylation measured by MassARRAY, the individual CpG methylation is shown on the right.The scheme above the graph represents the tested CGI (red line), in relation to the predicted transcription start site (green line), the exons (black boxes) and alternatively spliced exons (white boxes) of *adora2a*. Dashed lines indicate the 1500 bp up- and downstream region of the CGIs wherein the listed transcription factor binding sites are found. Boxplots below show the mean methylation in DCM and controls at the corresponding CGI. The pattern of CpG methylation as assessed by bisulphite-sequencing in multiple clones is shown for CGI-1 at the bottom of the figure.

As denoted above, we carried out an independent replication and fine mapping of the selected genes in a larger cohort of 30 idiopathic DCM patients and 28 controls. All selected candidates were fine-mapped by using MassARRAY (Ehrich et al, 2005). For each gene, several CpGs were retrieved and their methylation status quantified. From 20 candidate genes, 12 showed the same direction of altered methylation between the screening and the replication stage and four of them reached statistical significance, namely *LY75* (*p* = 0.000), *ERBB3* (*p* = 0.013), *HOXB13* (*p* = 0.001), and *ADORA2A* (*p* = 0.011). Figs 3 and 4 present the mean methylation changes of the replicated genes. Additionally, methylation of individual CpGs is displayed for *LY75* and *ERBB3* (Fig. 3), *HOXB13* (Fig. 4) and *ADORA2A* (Supporting Information Fig 1). Interestingly, *ADORA2A* showed significantly altered methylation throughout all tested CpGs, while *ERBB3* or *LY75* showed methylation alterations in a subset of the CpG nucleotides, possibly resulting in different functional consequences. Supporting Information Fig 2 gives the mean methylation of the remaining investigated CGIs. Since epigenetic marks may be also dependent on the gender, we additionally matched the gender ratio of cases and controls of the replication cohort to the ratio of the screening cohort, leading to significance of the same CGIs shown above. This is also true when matching females and males 1:1.

In addition to MassARRAY, we applied bisulphite sequencing for *LY75* and *ADORA2A* to fine-map and technically replicate our results. To do so, we generated 14 *LY75* and 12 *ADORA2A* clones from two DCM patients and two controls each, and sequenced them before and after treatment with bisulphite (Fig 3A and B; black circles = methylated CpG, white circles = unmethylated CpG). Bisulphite sequencing confirmed the corresponding MassARRAY data and, hence, demonstrated reliability of the latter technique.

**Figure 5:**
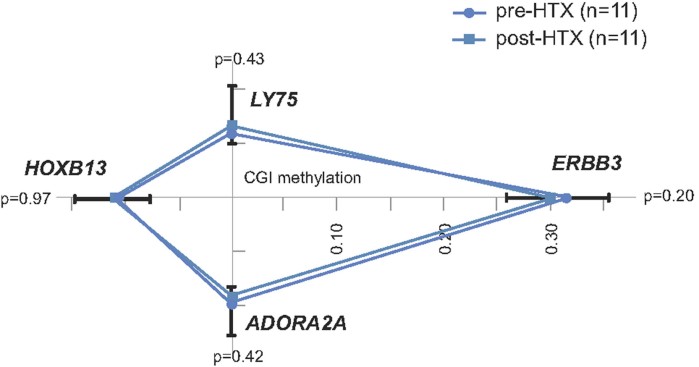
CpG island methylation of control subjects before and after heart transplantation The line diagram shows the highly similar degree of methylation of DNA derived from peripheral blood of control subjects pre- (light blue) and post- (dark blue) heart transplantation (*n* = 11) for *LY75, ADORA2A, ERBB3* and *HOXB13*. Error bars indicate standard deviation.

Next, to rule out a potential effect of the immunosuppressive medication received by the control subjects (Table 2) on the methylation of the investigated genes, we analysed the methylation patterns of genomic DNA from peripheral blood of 11 subjects drawn pre- and post-heart transplantation (HTX; mean timespan after HTX = 37 months under medication). As shown in Fig 5 we found highly comparable methylation levels (*p* = n.s.) of *LY75*, *ADORA2A*, *ERBB3* and *HOXB13* CGIs in pre- and post-HTX, indicating that the observed methylation differences in DCM are not due to methylation changes in the controls receiving immunosuppressive medication.

**Table 2:** Immunosuppressive medication of control individuals

Medication	*N*	%
Tacrolimus	19	54
Mycophenolat-mofetil	23	66
Ciclosporin	8	23
Prednisolon	11	31
Everolimus	14	40

All control individuals received one or more immunosuppressives.

### Differential gene expression and functional evaluation *in vivo* suggest contribution of *LY75 and ADORA2A* to DCM

DNA methylation is often correlated with changes in the accessibility of DNA to transcriptional activators, enhancers, or repressors. Hence, we first studied the impact of DNA methylation at the *LY75*, *ERBB3*, *HOXB13*, and *ADORA2A* loci on their gene expression by quantitative PCR (q-PCR) in controls, mild DCM (NYHA class II) and moderate to severe DCM (NYHA class III–IV).

**Figure 6:**
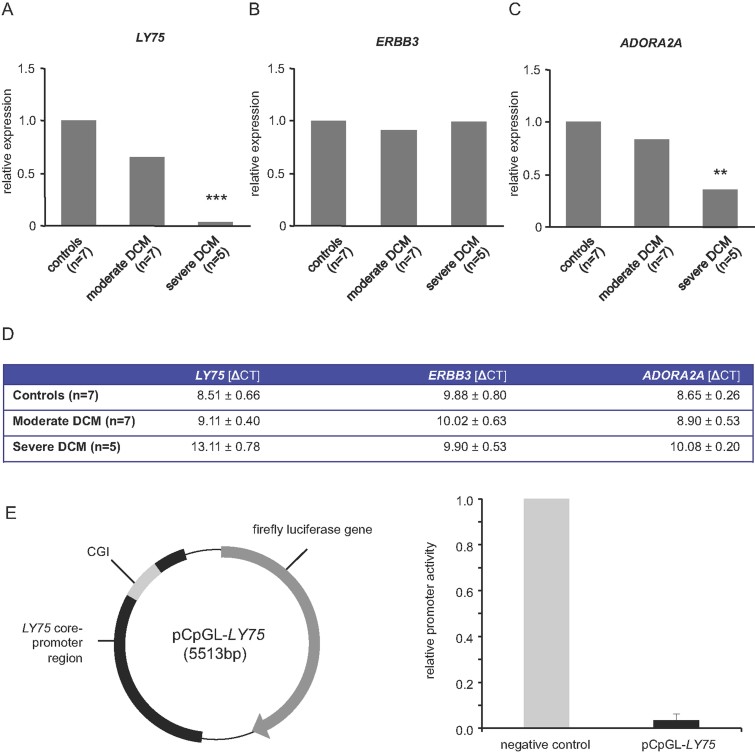
mRNA expression of genes with altered methylation status and gene promoter analysis **A–C.** Bar graphs showing relative mRNA expression levels of *LY75*, *ERBB3* and *ADORA2A* in mild and moderate-severe DCM in comparison to controls. (A) *LY75* expression is strongly reduced (relative expressio*n* = 0.04), while (B) *ERBB3* mRNA levels are not differentially regulated. (C) *ADORA2A* is also significantly down-regulated in DCM (relative expressio*n* = 0.37).**D.** Given are the mean delta-CT values (±SEM) of *LY75*, *ERBB3*, and *ADORA2A* in the different groups (controls, moderate DCM, severe DCM). The reference is based on the mean of the three housekeeper genes: *GAPDH*, *RPL13*, *β-actin*.**E.** Left: Schema of the vector construct to measure *LY75* promoter activity without and after treatment with the methylase *Sss*I. Right: Relative promoter activity (mean of three technical replicates ± SD) showing the strongly reduced activity in *LY75* after methylation, whereas the negative control does not show significant differences.

For *ADORA2A*, we found a positive relationship of gene expression with methylation (relative expressio*n* 0.33; *p* = 0.002), while *ERBB3* did not show significant alterations in cardiac expression levels (Fig 6B–D). *HOXB13* transcripts could not be PCR amplified in LV biopsies from patients and controls. In case of *LY75*, the hypermethylated CpG island is relatively close to the transcriptional start site (distance 1395 bp), whereas the distance between the CGIs in *ERBB3* (7518 bp) and *ADORA2A* (14,329 bp) is markedly larger (Figs 3 and 4). Since we also observed a strong reduction in *LY75* expression in myocardial tissue (relative expression 0.04; *p* = 0.001) in patients with DCM (Fig 6A and D), we asked if increased promoter methylation is directly responsible for decreased transcriptional activity. Hence, we performed a luciferase promoter assay with methylated and unmethylated *LY75* promoters. The CpG-free control reporter vector pCpGL-CMV/EF1 served as a negative control. As shown in Fig 6E, we found a strong reduction in promoter activity after *in vitro* methylation, supporting the functional relationship between *LY75* hypermethylation and reduced expression in human DCM.

Since we found a rather unusual positive relationship of *ADORA2A* gene expression and methylation, we first sought to identify the predominant isoform of *ADORA2A* in the human myocardium. By PCR, we found that the longer *ADORA2A* transcripts ENST00000337539 and ENST00000541988 are highly expressed in the heart. However isoform ENST00000417596, which is highly expressed in peripheral blood, is not significantly expressed in the heart. We then investigated potential repressor regions in the close vicinity of the tested CGIs. For the detection of transcription factor binding sites, the ENCODE transcription factor ChIP-seq data was used. We found for the aberrantly methylated CGI-1 two potential repressor sites (CTCF and NFKB), for aberrantly methylated CGI-2 one repressor site (CTCF) and for the unaltered CGI-3 one repressor site (Max). Interestingly, both hypomethylated CGIs (1 and 2) carry CTCF binding sites, which were recently identified as epigenetic key regulators in various other diseases.

**Figure 7:**
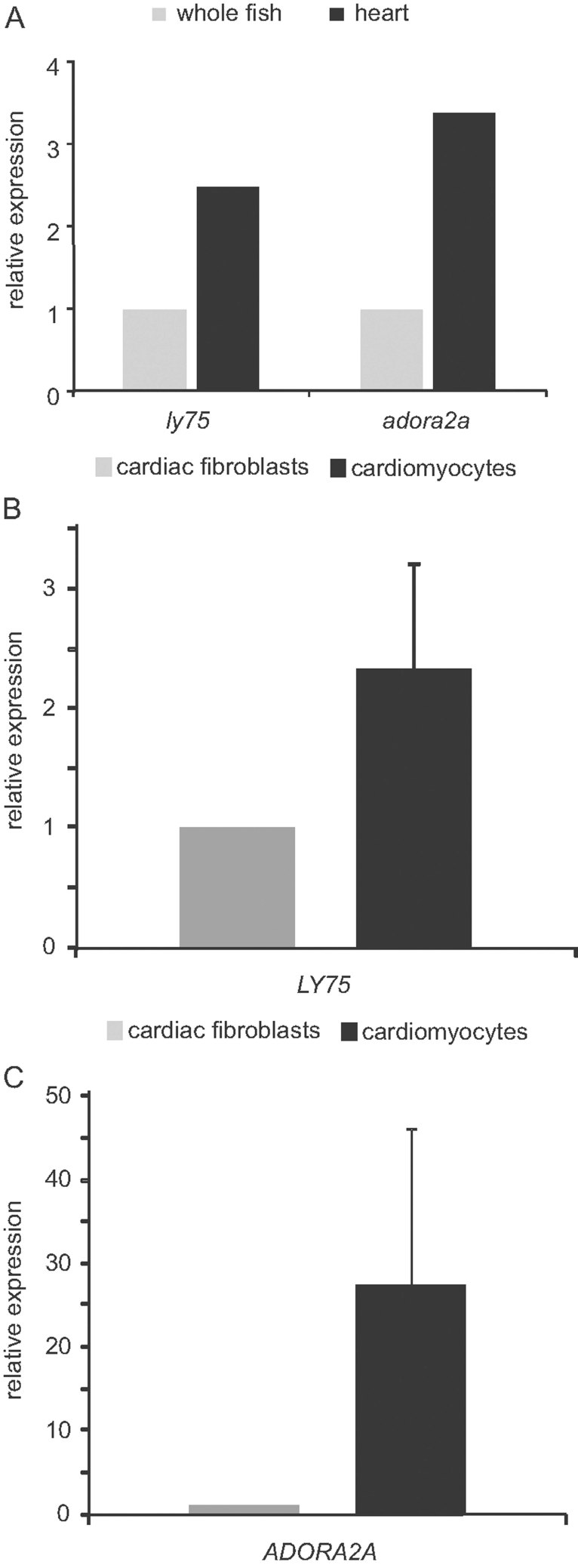
*ly75* and *adora2a* tissue and cell-type expression **A.** Relative expression levels of *ly75* and *adora2a* quantified by q-PCR in 40 whole zebrafish embryos (grey bars) and 200 isolated zebrafish embryonic hearts (black bars).**B, C.** Expression of *ly75* (B) and *adora2a* (C) in neonatal rat cardiomyocytes and cardiofibroblasts (*n* = 3 biological replicates). Error bars indicate standard deviation.

**Figure 8:**
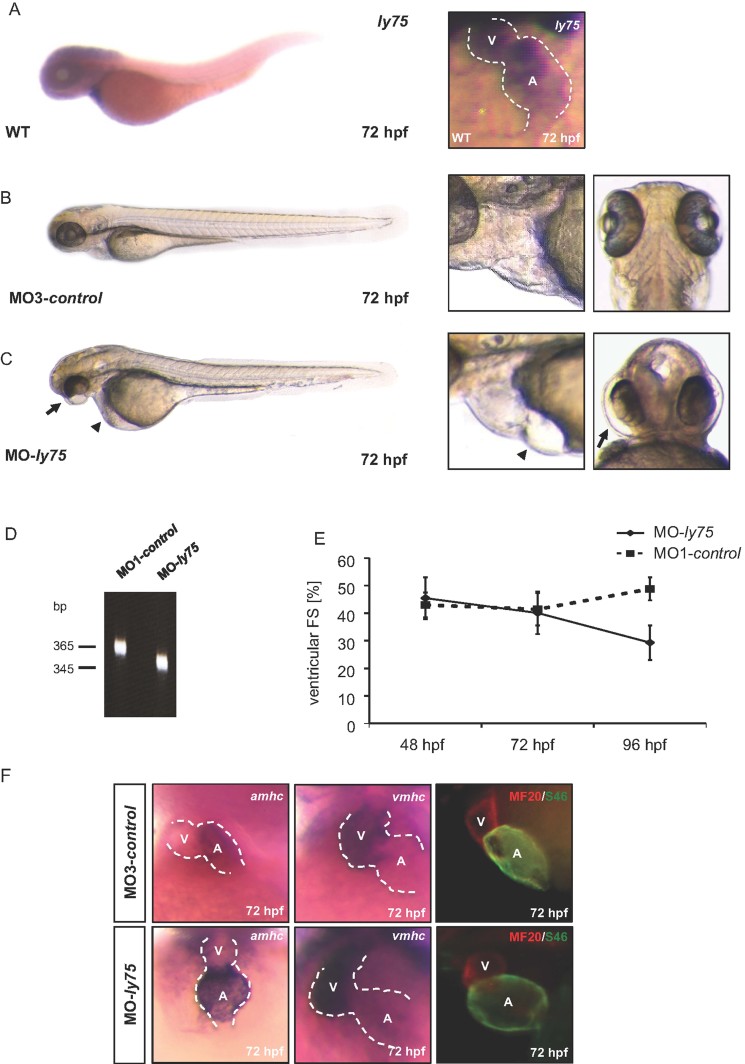
*ly75* is essential to maintain cardiac contractility. hpf, hours post fertilization; *amhc*, *atrial myosin heavy-chain*; *vmhc*, *ventricular myosin heavy-chain*; A, atrium; V, ventricle. Error bars indicate standard deviation **A.** mRNA antisense *in situ* hybridization showing that *ly75* is significantly expressed in the zebrafish heart.**B, C.** Lateral views of MO3-*control* (B) and MO-*ly75* (C) injected embryos at 72 hpf.**D, E.** After depletion of *ly75*, which can be monitored by cDNA splice-analysis (D), zebrafish hearts show reduced contractile force (E) and precardial blood congestion (arrowhead) as sign of manifest heart failure. *Ly75*-morphants also show noticeable skin detachment most pronounced in the head/eye region (C).**F.** Molecular chamber definition is not impaired in *ly75*-morphants compared to the control-injected zebrafish as demonstrated by regular *amhc*, *vmhc*, MF20 and S46 expression.

**Figure 9:**
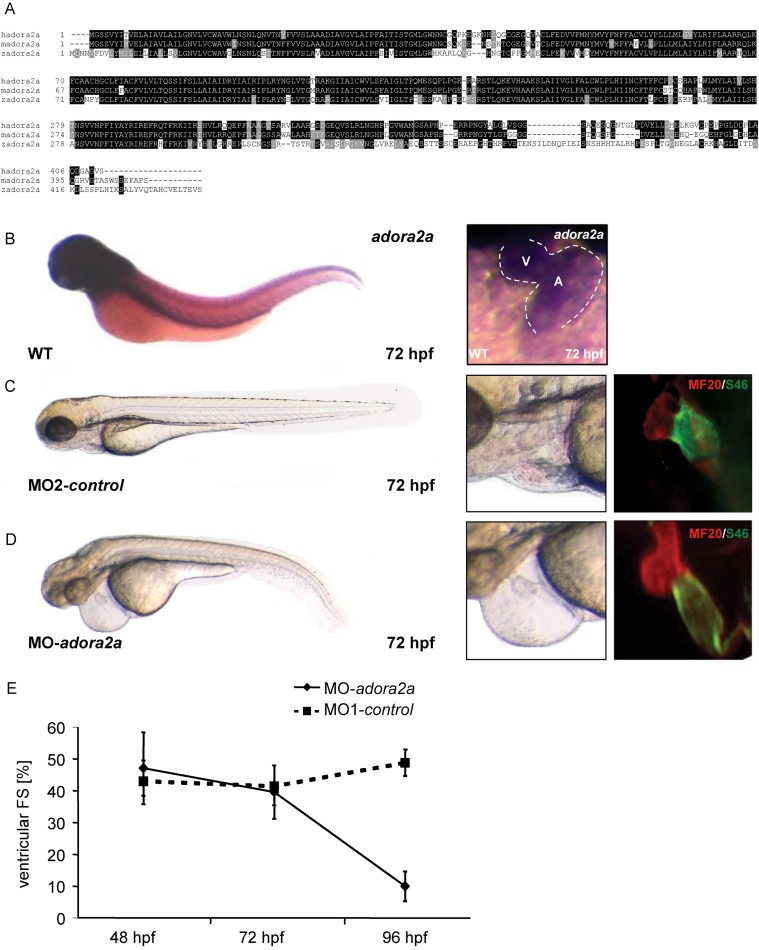
Knockdown of *adora2a* leads to severe heart failure in zebrafish **A.** Amino acid sequence alignments of human (h), mouse (m) and zebrafish (z) ADORA2A demonstrating its high cross-species homology. Black boxes indicate amino acid identity, grey boxes indicate amino acids with similar chemical properties.**B.** *Adora2A* is significantly expressed in the zebrafish heart.**C, D.** Lateral view of MO2-*control* (C), and MO-*adora2a* (D) injected embryos at 72 hpf.**E.** After injection of MO-*adora2a*, 66% of morphant embryos develop progressive heart failure and deterioration in ventricular contractility measured by fractional shortening at the indicated developmental stages. Error bars indicate standard deviation.

Since *LY75* and *ADORA2A* were not previously known to be involved in DCM or heart failure pathogenesis and both showed significant downregulation in the myocardium of DCM patients, we investigated their functional roles by gene knockdown in zebrafish embryos (Dahme et al, 2009). We identified orthologous sequences for human *LY75* and *ADORA2A* by BLAST searches against the zebrafish Genbank database. Protein sequence identity between the zebrafish and human version was 61% for *adora2a* (Fig 9A) and 37% for *ly75* (Supporting Information Fig 3). Similar to the human situation, we found *adora2a* and *ly75* to be highly expressed in the zebrafish heart using q-PCR and RNA antisense *in situ* hybridization (Figs 7–9). Additionally, as shown in cultured neonatal rat cells, both genes are higher expressed in cardiomyocytes than in cardiofibroblasts (Fig 7B and C). Hence, to recapitulate the downregulation of both genes as observed in the human heart, we inactivated them in zebrafish embryos by injection of Morpholino-modified antisense oligonucleotides directed against the splice donor site of *ly75* or the translational start-site of *adora2a*. While control-injected zebrafish (standard control Morpholino as well as scrambled control Morpholinos) embryos did not show any obvious phenotype, splice site Morpholino-mediated knockdown of *ly75* resulted in partial skipping of exon 8 (Fig 8D) and a consecutive frameshift that predictably leads to a premature stop of protein translation. As a result, *ly75*-morphants developed late-onset heart failure with dilation of the atrium (Fig 8C) and reduced ventricular contractility beginning at the 96 h developmental stage (FS = 46 ± 5% at 48 hpf and 29 ± 4% at 96 hpf; Fig 8E, Supporting Information Movie 1). Additionally, *ly75*-morphants showed a pronounced detachment and oedema of the skin especially noticeable in the eye and head region (Fig 8C). MO-*adora2a*-injected embryos also developed severe heart failure with progressively decreasing ventricular contractility as measured by fractional shortening (Fig 9E, Supporting Information Movie 2). In detail, the ventricular contractility of *adora2a*-morphants decreased from 47 ± 11% at 48 hpf to 39 ± 8% at 72 hpf. By 96 hpf, both heart chambers became almost silent. Occasionally, we also observed atrial fibrillation in *adora2a*-morphants and embryos developed excessive pericardial effusion and precardial blood congestion as a consequence of the reduced cardiac function. For both, l*y75*- and *adora2a*-morphants, we saw no alterations in molecular chamber specification and expression of atrial and ventricular myosin heavy chain genes (Figs 8 and 9 and Supporting Information Fig 4).

## DISCUSSION

Epigenetic mechanisms are increasingly recognized as causes and modulators of human disease. Most studies conducted so far have focused on cancer and yet only few have investigated the role of epigenetic mechanisms, such as DNA methylation, in cardiovascular disease (Movassagh et al, 2011). This is surprising since epigenetic mechanisms are thought to control key adaptive and maladaptive processes such as cardiac hypertrophy, fibrosis and failure (Backs et al, 2006, 2008). Here, we investigated DNA methylation patterns on a genome-wide level in myocardium from patients with idiopathic DCM and functionally unaffected hearts of patients who had received heart transplantation. We found and confirmed aberrant DNA methylation alterations in a number of CGIs, suggesting that DNA methylation is associated with cardiac function and may modulate phenotypic characteristics of idiopathic DCM.

There are several epigenetic mechanisms in eukaryotes and some have already been linked to DCM or heart failure. Firstly, chromatin modifications through ATP-dependent enzymes from, *e.g.* the SWI/SNF genes enable the cell to regulate the expression of distinct gene programs in organ development and adaptation (Ho & Crabtree, 2010). Secondly, histone modifiers such as histone acetyltransferases (HATs) or histone deacetylases (HDACs) are known to play a key role during development and in maintenance of cardiac function (Backs et al, 2011; Montgomery et al, 2008; Zhou et al, 2011). Cardiac specific loss of HDAC1 and HDAC2 for example results in cardiac arrhythmias and heart failure (Montgomery et al, 2007), while loss of HDAC3 function leads to cardiac hypertrophy (Montgomery et al, 2008). Thirdly, short non-coding microRNAs (miRNAs) broadly influence gene expression (Humphreys et al, 2005) and are increasingly recognized as valuable diagnostic (Keller et al, 2011; Meder et al, 2011a, b) and potential therapeutic targets (Gambari et al, 2011). DCM caused by deregulation of miRNAs such as miRNA-133a has been recently described in animal models (Chen et al, 2008; Meder et al, 2008; van Rooij et al, 2007). The current study now adds DNA methylation as a novel mechanism in the pathogenesis of DCM and heart failure.

DNA methylation in single genes and its contribution to disease onset or progression has been demonstrated in studies for hypertrophic cardiomyopathy (Meurs & Kuan, 2011). Furthermore, Movassagh et al (Movassagh et al, 2011) reported distinct epigenomic features in explanted human failing hearts, highlighting a potential role of DNA methylation also in end-stage heart failure. However, feasibility of the used methyl-DNA precipitation and sequencing is restricted by the relatively large amounts of required tissue, which is not easily available from living patients. Furthermore, handling of the tissue and time points of DNA isolation were quite different in controls and patients. Tissue specimens from explanted hearts of end-stage heart failure patients may be altered due to tissue handling and could have undergone secondary alterations because of degradation, missing oxygenation, innervation and arrest of pumping function (Talens et al, 2010). Also, the authors did not replicate their important findings, which were obtained in four patients, in an independent series of unrelated samples and by an alternative methodology. Therefore, we used a different approach, confirmed our findings in a large replication cohort with two alternative methodologies and applied functional studies to underscore the relevance of our results.

Since availability of appropriate myocardial tissue from living patients is limited, studies on epigenetic alterations in patients with heart disease are rare. As a primary referral centre for cardiomyopathies, we could enroll a high number of patients and controls in this study. Our controls are not completely healthy, but had previously received heart transplantation. Hence, potential confounders include patient's medication and medical history. Importantly, on the functional level all controls had an unconstrained cardiac function as a prerequisite and we could apply the exactly same standardized procedure to sample and process left ventricular biopsies from DCM patients and controls. However, all controls received immunosuppressive medications to prevent organ rejection. This medication might have an influence on at least some genes, *e.g.* glucocorticoids exert effects on DNA methylation of *Fkbp5* (Yang et al, 2012). As shown above, we found unchanged methylation levels for the tested genes in control subjects before and after heart transplantation, showing that immunosuppressives do not, at least in whole peripheral blood, impact on CGI DNA methylation of *LY75*, *ADORA2A*, *ERBB3* and *HOXB13*. However, it still cannot be completely ruled out that the medication exerts a different effect on cardiac tissue, which is a limitation of the current study.

Tissue samples are composed of different cell types, which might change during disease progression. In the present study, we have paid special attention to patient selection and the process of tissue sampling. Consequently, this resulted in high homogeneity of overall methylation patterns as shown above. In a study by Grzeskowiak et al it was demonstrated that differences in gene expression of LV myocardial biopsies from controls and idiopathic DCM patients were almost exclusively due to genes expressed in cardiomyocytes and that the contribution of the second most common cell type, fibroblasts, is rather low and mostly unchanged during DCM (Grzeskowiak et al, 2003). The increased fibrosis observed in human DCM, for instance, seems to be mainly due to extracellular collagen deposition rather than an increase of cardiofibroblasts. Our *in vivo* experiments clearly underline that reduced expression of *LY75* and *ADORA2A* in cardiomyocytes leads to heart failure as observed in our patients. Together, this approach allowed us to study epigenetic mechanisms and their functional consequences without the confounding background of organ hypoxia and apoptosis in explanted hearts of deceased patients.

Our global methylation analyses identified cardiac disease pathways and were complemented by extensive validation studies applying the highly precise MassARRAY and bisulphite sequencing techniques (Flotho et al, 2009; Ghoshal et al, 2010). We found 12 out of 20 genes showing the same direction of dys-methylation as found in the screening stage, and four reached statistical significance. This has at least three reasons: (i) we expected a high false positive rate due to the relatively small screening cohorts in contrast to the number of measured epigenetic features, (ii) we performed the replication measurements with a completely independent methodology, and (iii) we carried out these analyses in independent patients and controls. Therefore, the genes identified here represent consistently validated targets that show an epigenetic mode of regulation.

The deciphering of the functional implications of changes in DNA methylation is difficult but of pivotal importance. Since in many cases changes in CpG methylation are correlated with the accessibility of DNA to transcription factors and polymerases, measurements of mRNA levels represent an appropriate methodology. This was shown by using different model organisms such as fish (Mirbahai et al, 2011), mice (Tryndyak et al, 2011), as well as human tissues (Movassagh et al, 2010). In the present study, we find that methylation of novel cardiac genes is altered in patients with DCM. Hence, it can be hypothesized that the dys-methylated and dys-regulated genes also exert a functional role in the heart. The receptor tyrosine-protein kinase ERBB3 belongs to the membrane-bound epidermal growth factor receptor (EGFR) family. Functionally it is implicated in SOX10-mediated neural crest and early heart development (Prasad et al, 2011). Although we could not observe changes in ERBB3 transcript levels, the significant changes in DNA methylation could be a susceptibility factor mediated through yet unknown pathways, *e.g.* by exerting effects on DNA stability, ERBB3 isoform expression or histone binding. LY75, a collagen-binding mannose family receptor, is transcriptionally controlled by the interleukin-6 receptor IL6Rα and mediates antigen uptake and presentation in a clathrin-dependent manner (Giridhar et al, 2011; Tel et al, 2011). For DCM, a correlation of IL-6 levels and cross-linked type I collagen was found, potentially implicating a role of IL-6/LY75 signalling in cardiac remodelling (Timonen et al, 2008). For *LY75*, the dys-methylated CpGs reside within a classical CpG island covering exon 1 as well as part of the 5′ upstream region. Its transcriptional start site is predicted in very close vicinity (1395 bp). Our findings of significantly increased DNA methylation together with strongly reduced *LY75*-mRNA levels in DCM patients suggested a functional role. While it is to our knowledge not possible to recreate exactly the same methylation patterns *in vitro* as seen in the primary tissue, we could investigate here the functional consequence of global *LY75* promoter methylation, suggesting a direct link between methylation and transcriptional activity. We also knocked down *ly75* in the zebrafish model and found not only cardiac dysfunction in *ly75*-ablated embryos, but also a noticeable skin detachment phenotype, potentially due to disturbed collagen production. The adenosine receptor A2A (*ADORA2A*) is a member of the G protein-coupled receptor family and is highly abundant in neurons of basal ganglia, T lymphocytes, platelets and vasculature, but also shows high expression in myocardium as shown above. In the heart, activation of *ADORA2A* enhances cAMP production through αGs proteins (Sommerschild & Kirkeboen, 2000) and overexpression results in increased contractility and sarcoplasmic reticulum Ca^2+^ uptake (Hamad et al, 2010) as well as cardioprotection in ischemia and reperfusion damage (Urmaliya et al, 2009). Surprisingly, although *ADORA2A* methylation was reduced, we found mRNA levels to be significantly down-regulated. Such a positive relationship was described for other genes and is not fully understood in every case (Zhang et al, 2006). Our CGI annotation was based on the criteria by Takai and Jones (Takai & Jones, 2002), which define a CpG island as a nucleotide sequence of 200 bp or greater in length or with a GC content of 50% or more, or a ratio of observed *versus* expected CpGs of 0.6 or higher. In case of *ADORA2A*, the tested CGIs only met the second criterion and may therefore not be classical CGIs. For such sites, one explanation can be that hyper-methylation cannot only impede the binding of transcriptional activators but also can affect repressors of transcription. We therefore performed an *in silico* analysis of the promoter region of *ADORA2A* and found two binding sites for the transcription factor CTCF (CCCTC-binding factor) in close vicinity to both significantly hypo-methylated CGIs, but not within the single unaffected CGI of *ADORA2A*. CTCF is a unique insulator-binding protein (Bell et al, 1999; Felsenfeld et al, 2004; Gerasimova & Corces, 2001; West et al, 2002), which can act as transcriptional repressor by blocking distinct enhancer regions (Bell et al, 1999; Filippova et al, 1996; Gaszner & Felsenfeld, 2006; Lobanenkov et al, 1990; Ohlsson et al, 2001). Intriguingly, CTCF sites emerge as central players in regulatory networks linking gene regulation with epigenetic modifications (Bell & Felsenfeld, 2000; Ohlsson et al, 2001). Accordingly, our observed decrease in *ADORA2A* expression might be a result of enhanced binding of CTCF repressors due to CGI hypomethylation, which needs to be investigated in subsequent studies.

The detection of epigenetic mechanisms in human heart disease represents an attractive option to identify and dissect completely novel pathomechanisms. The validated targets from this study that have shown functional relevance are most likely modifiers of DCM rather than being independent disease causes. Therefore, it is reasonable to investigate their potential as diagnostic and therapeutic targets specifically in DCM but also in heart failure due to other causes. In the cancer field, epigenetic drugs have already entered the clinical arena (*e.g.* decitabine or azacitidine in the treatment of myelodysplastic syndromes), and methylation patterns are used as biomarkers to subtype and stage various cancers as a critical step towards a more effective personalized care (Coppede, 2011; Litzow, 2011). Hence, the genes identified here may be relevant druggable targets in DCM and heart failure and may aid in disease classification or risk stratification.

## MATERIALS AND METHODS

### Patients and controls

The study was conducted in accordance with the principles of the Declaration of Helsinki. All participants of the study have given written informed consent and the study was approved by the relevant ethics committees. DCM was diagnosed according to the guidelines of the World Health Organization (WHO; Richardson et al, 1995). Inclusion criteria for DCM cases were the presence of reduced left ventricular systolic function (left ventricular ejection fraction <45% assessed by echocardiography) in the absence of relevant coronary artery disease (CAD) as determined by coronary angiography. Patients with valvular or hypertensive heart disease, history of myocarditis, regular alcohol consumption or cardio-toxic chemotherapy were also excluded. The control biopsy specimens were obtained from patients who had received heart transplantation. All patients of the control cohort had successful cardiac transplantation more than 6 months ago with normal systolic and diastolic function and no evidence for relevant vasculopathy as judged by coronary angiography. Furthermore, all controls showed freedom from relevant acute or chronic organ rejection.

### Processing of left ventricular biopsies

Biopsy specimens were obtained from the apical part of the free left ventricular wall (LV) from DCM patients or cardiac transplant patients (controls) undergoing cardiac catheterization using a standardized protocol. Biopsies were washed with NaCl (0.9%) and immediately transferred and stored in liquid nitrogen until DNA or RNA was extracted. DNA was extracted using the DNeasy blood and tissue kit, total RNA using the RNeasy kit according to the manufacturer's protocol (Qiagen, Germany). RNA purity and concentration were determined using the Bioanalyzer 2100 (Agilent Technologies, Berkshire, UK) with a Eukaryote Total RNA Pico assay chip. Since a critical issue for the reliability of gene expression analysis is the quality of the RNA samples (Vermeulen et al, 2011), a RNA integrity number (RIN) >6 was defined as minimum requirement for further analyses.

### DNA methylation profiling and fine-mapping

For measuring methylation profiles, we used the Infinium HumanMethylation 27 BeadChip assay from Illumina with 1000 ng DNA per sample. The procedure followed the manufacturers standard workflow, starting with the bisulphite treatment of the sample DNA leading to a conversion of unmethylated cytosins to uracil, while 5-methylcytosines remain unchanged. After amplification and fragmentation of the bisulphite-converted DNA, they were hybridized to the Infinium BeadChips. DNA methylation data for DCM patients and controls have been deposited in NCBI's Gene Expression Omnibus (Edgar et al, 2002) and are accessible through GEO Series accession number GSE42510 (http://www.ncbi.nlm.nih.gov/sites/entrez?db=gds&term=GSE42510[Accession]&cmd=search). Fig 1A represents quantile normalized bead color signals (green and red) from 9350 consecutive probes on all further analysed arrays.

### DNA methylation validation by MassARRAY and bisulphite sequencing

DNA methylation was validated by the MassARRAY technique as previously described (Ehrich et al, 2005). Briefly, 400–500 ng genomic DNA was chemically modified with sodium bisulfite using the EZ methylation kit (Zymo Research) according to the manufacturer's instructions. The bisulfite-treated DNA was PCR-amplified by primers designed to cover the Infinium probes that showed differential methylation at each locus. The amplicons were transcribed by T7 polymerase, followed by T-specific-RNAase-A cleavage. The digested fragments were quantified by MALDI-TOF-based technique. The primer sequences are given in Supporting Information Table 1. DNA methylation standards (0, 20, 40, 60, 80 and 100% methylated genomic DNA) were used to confirm the unbiased amplification of the amplicons. For *LY75* and *ADORA2A* we additionally generated 14 and 12 clones, respectively, from two patients with DCM and two controls, each, and sequenced them before and after treatment with bisulfite. The methylation data was statistically analysed by Students *t*-test, or ANOVA, followed by Dunnett's multiple comparison.

### Promoter luciferase assay

To assess the causative link of CGI methylation within the promoter region of *LY75* with reduced mRNA expression observed in myocardial tissue, we performed a promoter luciferase assay essentially as described before (Smith et al, 2006). Therefore, we cloned a 1.6 kb large core fragment (Supporting Information Table 2) of the *LY75* promoter region including the analysed CGI into *Spe*I/*Bam*HI sites of the CpG-free luciferase vector pCpGL-basic. As negative control for the methylation effect we utilized the CpG-free control reporter vector pCpGL-CMV/EF1 (Klug & Rehli, 2006). Next, we treated both vectors with the methylase *Sss*I (New England BioLabs) or leaved them untreated, respectively. The methylation was performed at 10 mM Tris, pH 7.9, 50 mM NaCl, 10 mM MgCl_2_, 1 mM DTT and 160 µM SAM at 37°C for 1 h (Blesa et al, 2008). Transfection of human HEK293A cells with equal amounts of the *Sss*I-treated or untreated pCpG-*LY75* or pCpGL-CMV/EF1 vectors was carried out using Lipofectamin (Invitrogen). Additionally, we co-transfected these cells with the pGL4.74 [hRluc/TK) *Renilla* control vector as a reference (Promega). After 48 h, cells were lysed and luciferase and *Renilla* activity measured according to manufactures protocol (Dual-Luciferase Reporter Assay System, Promega). Results are expressed as relative luciferase activity (background substracted and normalized to *Renilla* reference; three technical replicates) of the methylated *versus* the unmethylated constructs.

### Promoter region prediction and transcription factor binding site analysis

We applied the Promoter 2.0 Prediction Software that predicts transcription start sites (TSS) of vertebrate Pol II promoters in genomic DNA to estimate the distance of the CGIs from the potential transcriptional start sites (Knudsen, 1999). For the detection of transcription factor binding sites, the ENCODE transcription factor ChIP-seq data was used. For each region (size of the CGI and 1500 bp up- and downstream), we selected in the maximum the three factors up- and three factors downstream with strongest observed binding.

### Hierarchical clustering

To generate a graphical representation of the most significantly dys-methylated cardiac genes in the screening stage and to detect patterns among the respective genes, hierarchical clustering was applied. Prior to the clustering, the patients were ranked for each gene according to the degree of methylation, *i.e.* the patient with the lowest degree of methylation for a certain gene received a rank of 1, while the patient with the highest degree of methylation received a rank of 17, since 17 samples have met filter criteria for the screening stage. Then, for all samples and all genes the pair-wise Euclidian distances were computed and a bottom-up clustering on these distances was carried out. All computations were carried out using the statistical programming language R (Team-RDC, 2008).

### Gene network analysis

To test whether genes with differential methylation patterns belong to a certain biological category, we carried out an unweighted gene set enrichment analysis (GSEA; Keller et al, 2007). In detail, we first sorted all genes according to the absolute value of the median distances between DCM patients and controls, generated a sorted list of genes where the most differentially methylated genes were on top while genes without differential methylation were located at the bottom of the list. If one gene was represented by multiple features on the biochip (different methylation sites), the median position of all replicates in the sorted list was computed. This sorted list was then used as input for GeneTrail (Backes et al, 2007). For each category, a significance value is computed by a dynamic programming approach (Keller et al, 2007) and all significance values of a certain category have been adjusted for multiple testing using Benjamini–Hochberg adjustment (Benjamini & Hochberg, 1995). The advantage of the applied cutoff-free biostatistical approach is that genes with *p* values of 0.049 and 0.051 are considered to be almost equally important in our approach while genes with larger values in the middle and at the end of the list are considered to be not of relevance. In contrast, classical over-representation analyses relying on cutoffs (usually 0.05) would consider genes with *p*-value of 0.049 of relevance while genes with *p* values of 0.051 would be considered to be non-relevant, having the same impact as genes with large *p* values.

**The paper explained**

PROBLEM:

Dilated cardiomyopathy (DCM) is one the most frequent heart muscle diseases. Although several factors including genetic mechanisms have been shown to cause DCM, we still find many cases unexplained and observe high phenotypic variability with respect to disease severity and prognosis. Epigenetic mechanisms are increasingly recognized as causes and modulators of human disease. Therefore, we studied genome-wide cardiac DNA methylation in DCM patients and controls to detect a possible epigenetic contribution to DCM.

RESULTS:

We detected distinct DNA methylation patterns in left ventricular heart tissue of DCM patients and reproduced the epigenetic mode of regulation for several genes with previously unknown function in DCM, namely *Lymphocyte antigen 75* (*LY75*), *Tyrosine kinase-type cell surface receptor HER3* (*ERBB3*), *Homeobox B13* (*HOXB13*) and *Adenosine receptor A2A* (*ADORA2A*). The results were verified by alternative techniques in a well-phenotyped and large independent cohort of DCM patients and controls. Furthermore, we were able to show the functional relevance for the contribution of the identified genes in the pathogenesis of heart failure by using zebrafish as an *in vivo* model.

IMPACT:

Our results hint at a novel layer in the pathogenesis of DCM and heart failure and will likely impact on the development of novel biomarkers and future therapeutic strategies.

### Transcriptomic analyses

Quantitative real-time PCR (q-PCR) was performed in order to measure expression of selected genes. Primers were designed using NCBI Primer-Blast and synthesized by Eurofins MWG Operon (Ebersberg, Germany). Sixty nanograms of total RNA extracted from biopsies of independent DCM patients (moderate: *n* = 7; severe: *n* = 5) and controls (*n* = 7) was reverse transcribed using SuperScript III first strand cDNA synthesis kit (Invitrogen). q-PCR was carried out according to standard protocols with the SYBR-Green method (Thermo Scientific) using an ABI 7000 system (ABI). Specificity of each primer-pair was monitored by dissociation curve analysis. Threshold cycle (CT) values were assessed in the exponential phase of amplification and the data were analysed using the delta-CT method. The mean value of the reference genes *GAPDH*, *RPL13* and *β-actin* was used as a reference. To identify the predominant cardiac isoform of *ADORA2A*, we performed PCR with the following primer-pair for both isoforms (ENST00000337539 and ENST00000541988): 5′-CTGTGACATGGAGCAGGAGC-3′ and 5′-GCTGTCGTTTGCCATCGGCCT-3′. To evaluate the expression levels of *ly75* and *adora2a* in the zebrafish heart and whole organism, we performed q-PCR with the following primer-pair for *ly75*: 5′-CATGGCCAGTTTCGATCCAT-3′ and 5′-CACCTGGGACTACACCTCCT-3′ and *adora2a*: 5′-TGCTGACCCAGAGCTCCATA-3′ and 5′-AGAGGCATCATCGCGATCTG-3′. Results are shown as relative expression values (normalization against the housekeeping gene *elf1a*). To analyze the expression of *ly75* and *adora2a* in different cell types, we cultured neonatal rat cardiomyocytes and cardiofibroblasts for 5 days and proceeded as described above. q-PCR was performed using the primer pairs 18S RNA 5′-GGACATCTAAGGGCATCAC-3′ and 5′-CCTCCGACTTTCGTTCTTGA-3′; *LY75* 5′-CACGGTCTGATGAGCTGTGT-3′ and 5′-ACGAACTGCAACCTGACCAT-3′; *ADORA2A* 5′-CTGGTCCTCACGCAGAGTTC-3′ and 5′-GCGAAGGGCATCATTGCAAT-3′. The methylation data was statistically analysed by Students *t*-test.

### Morpholino-mediated gene knockdown in zebrafish

The zebrafish experiments were performed under institutional approvals that conform to the Guide for the Care and Use of Laboratory Animals published by The US National Institute of Health (NIH Publication No. 85-23, revised 1996). Care and breeding of zebrafish (*Danio rerio*) were as described (Meder et al, 2011a, b). The following Morpholino-modified antisense oligonucleotides (GeneTools, USA) were designed: The *ly75* (ENSDARG00000053113) Morpholino targets the splice-donor site of exon 3 (MO-*ly75*: 5′-GTGATGAAACGCACACCTCTCCTGA-3′; scrambled control MO3-*control*: 5′-GTCATGAAAGGCACACGTCTGCTCA-3′), while *adora2a* (ENSDARG00000033706) was targeted at its start-site (MO-*adora2a*: 5′-CATTGTTCAGCATGGTGAGGTCGCT-3′; scrambled control MO2-*control*: 5′-CATTCTTCACCATCGTGACGTGGCT-3′). Morpholinos or control oligonucleotides (MO-*control*) were injected into 1-cell stage embryos as described before (Meder et al, 2009). To confirm the efficiency of splice-site Morpholinos, we analysed their target region by cDNA splice-site analysis (Meder et al, 2011a, b).

### Functional assessment and statistical analysis of cardiac function

Still images and high-resolution video microscopy movies were recorded and digitized with a Zeiss microscope/MCU II. The functional assessment of cardiac contractility was carried out as described before (Meder et al, 2009). Atrial and ventricular diameters were measured to calculate fractional shortening with the help of the zebraFS software (http://www.mederlab.com). Results are expressed as mean ± standard deviation. MF20/S46 stainings and mRNA antisense *in situ* hybridization was performed as previously described (Meder et al, 2009).

## Supplementary material

As a service to our authors and readers, this journal provides supporting information supplied by the authors. Such materials are peer reviewed and may be re-organized for online delivery, but are not copy-edited or typeset. Technical support issues arising from supporting information (other than missing files) should be addressed to the authors.
