# Supplementary material for: Alterations in cardiac DNA methylation in human dilated cardiomyopathy
Source: EMBO Mol Med. 2013 Jan 22;5(3):413–29. doi: 10.1002/emmm.201201553 (PMC3598081; doi:10.1002/emmm.201201553)
Supplement: Supplementary file 3 [file emmm0005-0413-SD3.pdf]

EMBO Molecular Medicine

Alterations in cardiac DNA methylation in human dilated cardiomyopathy

Jan Haas, Karen S. Frese, Yoon Jung Park, Andreas Keller, Britta Vogel, Anders M. Lindroth, Dieter Weichenhan, Jennifer Franke, Simon Fischer, Andrea Bauer, Sabine Marquart, Farbod Sedaghat-Hamedani, Elham Kayvanpour, Doreen Köhler, Nadine M. Wolf, Sarah Hassel, Rouven Nietsch, Thomas Wieland, Philipp Ehlermann, Jobst-Hendrik Schultz, Andreas Dösch, Derliz Mereles, Stefan Hardt, Johannes Backs, Jörg D. Hoheisel, Christoph Plass, Hugo A. Katus, Benjamin Meder

Corresponding author: Benjamin Meder, University Heidelberg

Review timeline:

Submission date:	08 May 2012
Editorial Decision:	08 June 2012
Revision received:	04 October 2012
Editorial Decision:	06 November 2012
Revision received:	15 November 2012
Additional Editorial Correspondence:	20 November 2012
Additional Author Correspondence:	27 November 2012
Accepted:	29 November 2012

Transaction Report:

(Note: With the exception of the correction of typographical or spelling errors that could be a source of ambiguity, letters and reports are not edited. The original formatting of letters and referee reports may not be reflected in this compilation.)

1st Editorial Decision 08 June 2012

Thank you for the submission of your manuscript "Alterations in cardiac DNA methylation suggest a role of epigenetic mechanisms in human dilated cardiomyopathy" to EMBO Molecular Medicine and please accept my apologies for the delayed reply. We have now received the reports from the three referees whom we asked to review your manuscript. You will see that they find the topic of your manuscript potentially interesting. However, they also raise significant concerns on the study, which should be addressed in a major revision of the manuscript.

Importantly, Reviewer #1 highlights potential biases in the study that should be discussed. In addition, Reviewer #3 highlights that zebrafish control pictures are highly similar to figures from a previous publication and to each other, which should be clarified. Importantly, both Reviewers #1 and #3 note that it is crucial to directly link altered gene expression and DNA methylation for the identified genes

Given the balance of these evaluations, we feel that we can consider a revision of your manuscript if you can convincingly address the issues that have been raised within the time constraints outlined below.

However, we realize that addressing all of the referees' criticisms might require a lot of additional time and effort and could be technically challenging. I am uncertain whether you will be able (or willing) to return a revised manuscript within the 3 months deadline and I would also understand your decision if you chose to rather seek rapid publication elsewhere at this stage.

Should you decide to embark on such a revision, revised manuscripts should be submitted within three months of a request for revision. They will otherwise be treated as new submissions. Should you find that the requested revisions are not feasible within the constraints outlined here and choose, therefore, to submit your paper elsewhere, we would welcome a message to this effect.

Yours sincerely,

Scientific Editor
EMBO Molecular Medicine

***** Reviewer's comments *****

Referee #1 (Comments on Novelty/Model System):

As indicated in the remarks to the authors, the model chosen might be biased due to specific medical treatment in the control and/or the cases groups. Suggestion to circumvent this bias is made in the report.

Referee #1 (Other Remarks):

Haas et al. presents the second attempt to map the whole genome methylation profile in heart failure vs normal heart. Due to major technical breakthroughs allowing the detection of whole-genome epigenetic modification, epigenetics is an emerging and important field, and could help enlarge our understanding of disease pathophysiology. Compared to the first published study, the present one address the problem of the effects of DNA methylation modifications associated with heart failure in a larger sample of patients and controls is using a different methodology and reports functional analysis to validate the potential effects of the identified epigenetically regulated genes. Overall the experimental plan and the bench work are well done, however a major shortcoming is related to the selection of the samples included in the study which might lead to a systematic bias in the result as detailed below.

Major concerns

1- Previous reports link transplant success to epigenetic modulation (McCaughan and al. Transplantation 2012). Such data indicate a potential major bias in the control population used in the paper, with hypermethylation possibly associated with engraftment success. Another bias could come from the effect of transplantation associated medication (i.e. immunosuppressants, or prior transplantation therapeutics in "controls") that could interact with epigenetic modification. Due to probable interactions of pharmacologic compounds with epigenetic modifications, it would be of interest to determine medical treatments of the cases and controls and have an evaluation/discussion of the possible interactions. An additional control group composed of un-transplanted normal heart should be included in the study to exclude these strong biases.
2- Both LY75 and ADORA2A are down-regulated at the mRNA level in DCM samples. However, one is hypermethylated and the other is hypomethylated. As hypothesized in the discussion, an inhibitory factor with reduced binding to the ADORA2A promoter would explain this discrepancy. However the authors did not present any evidence that this could be the case, neither experimentally nor in silico, (such as by identifying any potential cardiac repressor responding elements and associated methyl-dependent differences in the binding or transcriptional modulation of a reporter gene).
3- The two replicated genes were analyzed through MO mediated knock-down in zebrafish. LY75 and ADORA2A both lead to heart failure when abolished in fish. Are they both expressed in heart tissue? The results presented in fig4 do not permit estimation of the relative expression compared to reference genes (DeltaCt) in heart samples. Is this true in zebrafish as well? Supposing they are expressed at a level compatible with a functional role in heart (a prerequisite to support the claim for their implication in epigenetic modulation of heart failure) it is not surprising that they could lead to cardiac phenotype when extinguished. From their past experience with zebrafish MO and to support the data the authors could maybe present an estimate of the frequency of the association between the

knocking-down of a cardiac expressed gene and a cardiac phenotype in zebrafish. This would help estimating the specificity of this functional assay.

Minor points:

1- The authors started the paper presenting DCM as "one of the most frequent heart muscle diseases". Is this statement taking into account ischemic forms of DCM? According to the material and methods, the definition of DCM retained for selection of patients is more restrictive and fits with an idiopathic DCM definition, a condition which excludes ischemic DCM and is not responsible "for about 30-40% of all heart failure" as its prevalence is 1:2500. The authors should comment on that point, and specify which disease they are studying.
2- In figure 1, clearer indication and discussion is required to explain why some dark dots are highly methylated in cases or in controls.
3- The presentation of the results in fig1b might be modified to take into account that the 3 groups (NIA? Please spell out) are probably not independent. The "pathological conditions" probably includes a large part of the two others and the sum of the dys-methylated hits of both groups (337+359=696) is far more than the overall 428 CpG (or genes?). The authors could use a Venn diagram.
4- In the results section it is not clear to the reviewer what is presented in fig2C. What is the difference between "dysmethylated genes" and those presented in fig 2a and b? Why are the error bars so drastically different between fig 2a vs b & c?
5- The IDCM phenotype described in the materials and methods section does not indicate that cases due to excess alcohol consumption were also excluded. Please indicate if this is the case and if not how it could affect epigenetic modification.

Referee #2 (Comments on Novelty/Model System):

This is a good model system - (Zebrafish) - for testing the roles of specific genes in cardiac development. One could argue for a mouse model of "real dilated cardiomyopathy", but I think this could be done in a future paper and is not needed for the current report.

Referee #2 (Other Remarks):

This is a well executed and (nearly) complete study of DNA methylation in human dilated cardiomyopathy. The authors used Illumina 27K methylation BeadChips as their primary screening platform, followed by MassArray for validations. They included a replication set of biological samples, and did functional knockdown experiments in Zebrafish. The figures are clear and easy to understand, so the study will be easy to repeat in other labs.
The Discussion is also well written, although I would suggest avoiding the word "unfortunately" in their criticism of previous studies (just stating the prior findings and the experimental differences would be sufficient).
I have one request for more data, namely, that the authors should carry out standard bisulfite sequencing (with cloning)on their two best "hits" (ADORA and LY75)in 2 cases and 2 controls, to show the precise PATTERNS of CpG methylation and the range of heterogeneity of these patterns within the DCM and control cardiac tissue (which will be evident from sequencing and showing multiple clones).

Referee #3:

In this study, Meder et al. analyse changes in DNA methylation in patients with dilated cardiomyopathy (DCM). Two cohorts of DCM patients and controls were investigated and DNA methylation was analyzed by Illumina bead chip array and MassARRAY techniques. In the first cohort, 90 CpGs were differentially methylated. Validation in a second cohort finally resulted in 4 CpG regions which were differentially methylated. These CpGs were associated with the genes LY75, ERBB3, HOXB13 and ADORA2A. For two of these genes, mRNA expression levels were

different between control and DCM hearts. LY75 showed an inverse relation between DNA methylation and expression. For ADORA2A, methylation and expression were both reduced in DCM hearts. Finally, the authors injected morpholinos targeting LY75 and ADORA2A into zebrafish and demonstrate that this led to heart failure in the fish model.

While the topic is of general interest and the findings are novel, three crucial points should be addressed:

1) A direct causal link between DNA methylation and gene expression should be demonstrated for LY75 and ADORA2A. This is particularly important to rule out that two differentially methylated genes which were derived from an initial screening method using 27000 CpG probes on the Illumina array did not show differential methylation and expression by chance (details in 14) below).

2) As the heart is a complex tissue and its composition changes with disease, the cell type(s) involved in differential methylation and expression of LY75 and ADORA2A should be identified (details in 15) below).

3) The duplication of fish images in figures 5A, 6A (this manuscript) and figure 1A in Meder et al. 2009 and the respective control measurements should be clarified (details in 10) below).

Further specific questions should be addressed:

4) Results, page 5, line 11: The data derived from the first cohort were tested with an unadjusted p-value. It would be correct to use adjusted p-values. The results should be modified accordingly.

5) Results, page 6, line 3: It remains unclear whether the data displayed in figure 2 are derived from the first cohort or from the second cohort. All figure legends should clearly indicate from which cohort the data are derived and what the numbers of samples per group are. With respect to figures 2A and 2B, it is unclear why the error bars are so different between the two groups - barely visible in 2A and relatively large in 2B. The authors should rule out a systematical error and comment on this effect. Where the detection values for the methylation probes on the array identical for both groups?

6) Figure 2C: If the results displayed here show "the 20 most significantly dysmethylated genes" where is LY75? Are ADORA2A, ERBB3, HOXB13 significantly differentially methylated? Please give p-values, number of samples per group, which cohort etc.

7) Results, page 6, line 6 from bottom: Figure 3 is supposed to show methylation of "each of the successfully replicated genes". However, it remains unclear why the results for HOXB13 are not displayed? Please show these methylation results.

8) Figure 4: Please label the y axes of this figure.

9) Figure 4: Why did the authors separate gene expression for two groups of DCM patients, moderate and severe DCM, but did not perform the same analysis for DNA methylation? Methylation results should be shown for the same two DCM groups and it should be tested whether DNA methylation and gene expression correlate significantly in a regression analysis.

10) Figure 5 & 6: The zebrafish pictures shown in 5A and 6A are identical. Also the functional data for "MO controls" displayed in figures 5D and 6D are identical. This should be clearly stated in both figure legends. In order to find out, wether only one control experiment has been performed, the reviewer finds one paper referenced in the methods section (Meder et al., 2009) which also shows the same control fish (figure 1A) - however with different functional results. The authors should thoroughly address how this could happen and clarify how many control experiments were performed for the present study and which control oligonucleotides were actually used.

11) The exact identity of the CpGs which are displayed in the manuscript should be given in a supplementary table. All array results should be deposited in a public database.

12) Page 31, table 1: The percentage of female and male samples differs between the groups in both

cohorts. As DNA methylation is subject to sex-specific imprinting, the groups should be adjusted to rule out gender effects on methylation.

13) Suppl. figure 1: The legend should be revised as for most CpGs not even a clear trend for differential methylation is visible. Thus, it would be sufficient to point out HOXB13 as significantly differentially methylated. Please also correct the two different p-values in the legend.

14) At the end of this study two genes remain which show differential methylation, one up and one down, in DCM while both are expressed at lower levels in severe DCM. It would be important to demonstrate that altered gene expression and DNA methylation are indeed directly linked with each other and that ADORA2A and LY75 are not just by chance occuring on both lists. This is particulary important as both genes show opposite alterations in DNA methylation levels despite reduced mRNA expression.

15) ADORA2A and LY75 show very low expression levels in the heart and cardiac myocytes (based on www.biogps.org (ADORA2A, LY75) and www.proteinatlas.org (for LY75)). However, their expression levels are many-fold higher in other cell types, including immune cells. As the cellular composition of healthy and diseased hearts may differ dramatically and DNA methylation is supposed to be very cell type-specific, it would be important to identify the cell type(s) in the heart which are associated with the observed differences in methylation and expression.

1st Revision - authors' response 04 October 2012

Referee #1

1) Due to probable interactions of pharmacologic compounds with epigenetic modifications, it would be of interest to determine medical treatments of the cases and controls and have an evaluation/discussion of the possible interactions. An additional control group composed of untransplanted normal heart should be included in the study to exclude these strong biases.

We have now evaluated the medical treatment of all controls included in this study (see table 2 of the revised manuscript) and discuss known interactions with epigenetic mechanisms. We also agree with the reviewer that the evaluation of the interaction of these therapies with epigenetic alterations of our identified targets would be valuable. Hence, we performed important experiments and find unchanged methylation levels for the dys-methylated genes in control subjects before and after receiving heart transplantation (corresponding to the situation without and with immunosuppressive medication). These new data show that immunosuppressives do not impact on DNA methylation of *LY75, ADORA2A, ERBB3* and *HOXB13* CGIs. We included these information on manuscript pages 7, 12, in table 2, and figure 5.

2) Both LY75 and ADORA2A are down-regulated at the mRNA level in DCM samples. However, one is hypermethylated and the other is hypomethylated. As hypothesized in the discussion, an inhibitory factor with reduced binding to the ADORA2A promoter would explain this discrepancy. However the authors did not present any evidence that this could be the case, neither experimentally nor in silico.

First, we now have identified for the first time the predominantly expressed isoforms of *ADORA2A* in the human heart (ENST00000541988, ENST00000337539). Next, we performed additional MassARRAY experiments for 2 further *ADORA2A* CGIs to identify potential patterns of epigenetic regulation. Interestingly, as shown in figure 4B and on page 7 in the revised manuscript, we found 1 additional, hypomethylated CGI within *ADORA2A* (CGI-2). Next, we performed the suggested *in silico* TF binding site analysis. Amongst others, we found two strong binding sites for the transcription factor CTCF (CCCTC-binding factor), which is the only significant Insulator-binding protein identified in vertebrates (Bell et al, 1999; Felsenfeld et al, 2004; Gerasimova & Corces, 2001; West et al, 2002). CTFC has been shown to act as repressor of transcription by its enhancer blocking insulator activity (Bell et al, 1999; Filippova et al, 1996; Gaszner & Felsenfeld, 2006; Lobanenkov et al, 1990; Ohlsson et al, 2001).

From the three different CGIs within *ADORA2A*, two showed hypomethylation. In both cases, CTFC binding sites have been identified next to these CGIs. Assuming CTCF to act as an enhancer blocking transcription factor in both cases could explain the down-regulation of mRNA levels together with the observed CGI hypomethylation. Additionally, we added to the discussion the fact that these CTCF sites are prototypes for epigenetic-genetic interaction mechanisms in different diseases (manuscript page 15-16). For *LY75* we now have cloned its promoter and performed luciferase promoter assays to dissect the effect of CGI methylation on *LY75* transcription. These data that link hypermethylation of *LY75* with its down regulation can be found in figure 6E.

3) LY75 and ADORA2A both lead to heart failure when abolished in fish. Are they both expressed in heart tissue? The results presented in fig4 do not permit estimation of the relative expression compared to reference genes (DeltaCt) in heart samples. Is this true in zebra fish as well?

We have comprehensively addressed this issue. First, we give the DeltaCT values for our real time PCR experiments in human LV biopsies (see updated figure 6D), showing significant expression of *ADORA2A* and *LY75* in the heart. Secondly, we show that *ADORA2A* and *LY75* are highly abundant in the zebra fish heart (Fig. 7A) by q-PCR as well as mRNA-antisense *in situ* hybridization (Fig. 8A and 9B).

4) From their past experience with zebra fish MO and to support the data the authors could maybe present an estimate of the frequency of the association between the knocking-down of a cardiac expressed gene and a cardiac phenotype in zebra fish. This would help estimating the specificity of this functional assay.

This is a more difficult estimation, since there are to our knowledge no systematic studies on that topic. However, from our own work we functionally and molecularly evaluate approximately 15 novel genes per year in the zebra fish. From this already biased selection (e.g. genes that are expressed in the heart), only the minority leads to a specific cardiac phenotype. Examples described before include e.g. Hippe et al., 2009, where the injection of an NDPK A Morpholino did not result in a cardiac phenotype.

To further underline the specificity of our experiments, we now also performed MO knock-down experiments with scrambled Morpholinos against *LY75* and *ADORA2A*. Here, the same sequence was used as for the gene knockdowns with exception of 5 scrambled bases (see table below). These injections with the same amounts of MO did not result in a cardiac abnormality (see Fig. 8 and Fig. 9 of the revised manuscript). We now also undertook experiments were severe developmental defects can be excluded (updated figure 8 and 9).

Gene	Morpholino sequence	Scrambled control/5-mis-pair control
LY75	5'-GTGATGAAACGCACACCTCTCCTGA 3'	5'-GTCATGAAAGGCACACGTCTGCTCA-3'
ADORA2A	5'-TTGTTCAGCATGGTGAGGTCGCT-3'	5'-CATTCTTCACCATCGTGACGTGGCT-3'

5) The authors started the paper presenting DCM as "one of the most frequent heart muscle diseases". Is this statement taking into account ischemic forms of DCM?
We clarified this statement at several passages in the manuscript (e.g. page 3) and now indicate that we mean primary heart muscle diseases, but not ischemic forms.

6) According to the material and methods, the definition of DCM retained for selection of patients is more restrictive and fits with an idiopathic DCM definition, a condition which excludes ischemic DCM and is not responsible "for about 30-40% of all heart failure" as its prevalence is 1:2500. The authors should comment on that point, and specify which disease they are studying.

© EMBO

We have addressed that point in the revision to avoid confusion. The number of 30-40% of heart failure cases comes from different studies on heart failure including large-scale clinical trials. There, about 30-40% of the study population was regarded to as non-ischemic dilated cardiomyopathy. However, this might be an overestimation since they also included patients after myocarditis etc. Please see our revised statement on DCM on page 3 (introduction) of the manuscript.

7) In figure 1, clearer indication and discussion is required to explain why some dark dots are highly methylated in cases or in controls.

Each dot in the figure corresponds to one CpG island. Shown on the x-axis is the mean value of the respective CpG island in DCM patients, on the y-axis the mean in controls. The red dots correspond to a significant up-regulation in DCM patients while the green dots correspond to a significant down-regulation in patients. The colored dots show a strong tendency to be those being furthest away from the bisecting line, i.e. they are most dys-regulated while points on the bisectrix have the same value in DCM patients and controls. However, some of the points that are rather far away from the bisecting line are not colored (= not significantly dys-regulated) although they have high fold changes. In the hypothesis test, besides the information of how differentially methylated the patient and control cohort is, also the standard deviation is used, meaning that these respective CpG islands show usually high variance. We added a respective sentence to the manuscript.

8) The presentation of the results in fig1b might be modified to take into account that the 3 groups (NIA? Please spell out) are probably not independent. The "pathological conditions" probably includes a large part of the two others and the sum of the dys-methylated hits of both groups (337+359=696) is far more than the overall 428 CpG (or genes?). The authors could use a Venn diagram.

The NIA is the National Institute of Aging, we added this to the revised version of the manuscript. Of course, the referee is fully right in that there is an overlap between the three disease gene sets. Following the advice of the referee we modified Figure 1C of the revision. Now, we provide a 3-Way area proportional Venn diagram such that the reader can immediately recognize that there is an overlap and how substantial this overlap is. We applied a standard unweighted GSEA where all CpG islands are taken into consideration, the full list of CpG islands is sorted according to the p-value and used as input for the respective approach. The advantage of this approach is, in comparison to approaches where only significant genes are considered, a gene with a p-value of 0.049 and a gene with p-value of 0.051 are treated almost the same while classical over-representation analyses would consider the first gene while omitting the second. We pointed this out more strongly in the Materials section of the revised manuscript.

9) In the results section it is not clear to the reviewer what is presented in fig2C. What is the difference between "dysmethylated genes" and those presented in fig 2a and b? Why are the error bars so drastically different between fig 2 a vs b & c?

We apologize for any confusion. Figure 2 A and B represent the CpGs, which are most strongly altered in their methylation-status (absolute value change). Figure 2 C represents CpGs that are most significantly altered in their methylation-status. We corrected any misleading description in the manuscript and removed an error resulting in a wrong selection and display of genes in Fig. 2 A and B. We truly apologize for that error.

10) The IDCM phenotype described in the materials and methods section does not indicate that cases due to excess alcohol consumption were also excluded. Please indicate if this is the case and if not how it could affect epigenetic modification.

We excluded patients with secondary causes of DCM, since as the reviewer mentions these factors might also impact on epigenetic regulation. Patients with regular or excessive alcohol consumption were also excluded. We modified the materials & methods section accordingly.

Referee #2

1) I have one request for more data, namely, that the authors should carry out standard bisulfite sequencing (with cloning) on their two best "hits" (ADORA and LY75) in 2 cases and 2 controls, to show the precise PATTERNS of CpG methylation and the range of heterogeneity of these patterns within the DCM and control cardiac tissue (which will be evident from sequencing and showing multiple clones).

We thank the reviewer for the good suggestion and now measured bisulfite methylation patterns from 2 DCM patients and 2 controls with 14 (*LY75*) and 12 (*ADORA2A*) individual clones, each. This bisulfite-sequence pattern of *LY75* and *ADORA2A* clearly resembles the MassARRAY and Infinium-array data (Figure 3A and 4B). Hence, our results are now replicated in 2 completely different cohorts and by 3 independent methods.

2) The Discussion is well written, although I would suggest avoiding the word "unfortunately" in their criticism of previous studies (just stating the prior findings and the experimental differences would be sufficient).

The reviewer is right and we changed our phrasing.

Referee #3:

1) The data derived from the first cohort were tested with an unadjusted p-value. It would be correct to use adjusted p-values. The results should be modified accordingly.

The referee addresses a general problem of high-throughput omics studies. Described as the curse of dimensionality or p>>n problem, the sampling density exponentially depends on the dimensionality of the feature space. In case of high-throughput discovery studies where easily tens of thousands of features are measured, however, due to the experimental cost and availability of the biological samples only a restricted screening cohort can be measured and even highly significant effects vanish after adjustment, especially if conservative approaches as Bonferroni adjustment are carried out. Basically, adjusted p-values mean to have fewer false positive candidates, at the cost of loosing true positive candidates. We decided to perform a funnel-like, two-staged strategy to account for false positives while not losing the true positives: 1) we screened for methylation differences on a genome-wide level and analyzed the data without adjustment for multiple-testing and 2) to detect the false-positives, we performed an independent replication study with 22 CpG Island (20 genes) in 58 individuals. The fact that the replication measurements were performed in an independent cohort with now two independent methods (MassARRAY and bisulfite-sequencing) shows the consistency of our identified targets, underlined by our functional data.

2) It remains unclear whether the data displayed in figure 2 are derived from the first cohort or from the second cohort. All figure legends should clearly indicate from which cohort the data are derived and what the numbers of samples per group are.

We have changed the figure legend 2, which now indicates that the data are derived from the screening cohort and also give the number of patients in each group for all figures (see figure 1-6).

3) With respect to figures 2A and 2B, it is unclear why the error bars are so different between the two groups - barely visible in 2A and relatively large in 2B. The authors should rule out a systematical error and comment on this effect.

The reviewer is right, there was an error leading to incorrect calculation of the most strongly dysregulated genes and their standard deviations. We have corrected this issue and apologize for the error.

4) Where the detection values for the methylation probes on the array identical for both groups?

The intensity distributions across all hybridizations on the BeadArray were highly similar. As shown in the bar plots in revised figure 1A, we see similar distributions in both color channels for DCM patients and controls.

5) Figure 2C: If the results displayed here show "the 20 most significantly dysmethylated genes" where is LY75? Are ADORA2A, ERBB3, HOXB13 significantly differentially methylated? Please give p-values, number of samples per group, which cohort etc.

As noted above, we relied on a two-staged study design to avoid the problems with multidimensionality. Hence, the candidates were selected according to their unadjusted p-value, rank in absolute methylation difference, or a combination of the two. This approach resulted in consistently replicated candidates and our new data also show the reproducibility by a third, independent method, namely bisulfite-sequencing. For your overview, we show below all relevant statistical data for your convenience.

	Screening cohort	Replication cohort
ADORA2A	p-unadj=0.001	CpG1: p=0.005
	n=9 cases, 8 ctrls	CpG2: p=0.016
		CpG3: p=0.027
		CpG4: p=0.027
		CpG5: p=0.024
		n=30 cases, 28 ctrls
LY75	p-unadj=0.003	CpG1: p=0.014
	n=9 cases, 8 ctrls	CpG2: p=0.438
		CpG3: p=0.005
		CpG4: p=0.168
		CpG5: p=0.003
		CpG6: p=0.093
		CpG7: p=0.011
		CpG8: p=0.298
		CpG9: p=0.00001
		CpG10: p=0.011
		n=30 cases, 28 ctrls
ERBB3	p-unadj=0.001	CpG1: p=0.003
	n=9 cases, 8 ctrls	CpG2: p=0.687
		CpG3: p=0.018
		CpG4: p=0.017
		CpG5: p=0.022
		CpG6: p=0.202
		CpG7: p=0.504
		n=30 cases, 28 ctrls

© EMBO

HOXB13	p-unadj=5.3E-05<br>n=9 cases, 8 ctrls	CpG1: p=0.133<br>CpG2: p=0.483<br>CpG3: p=0.109<br>CpG4: p=0.439<br>CpG5: p=0.621<br>CpG6: p=0.002<br>CpG7: p=0.341<br>n=30 cases, 28 ctrls

6) Results, page 6, line 6 from bottom: Figure 3 is supposed to show methylation of "each of the successfully replicated genes". However, it remains unclear why the results for HOXB13 are not displayed? Please show these methylation results.

This was due to constraints in the available figure space. As requested, we now added the methylation data of *HOXB13* in figure 4A.

7) Figure 4: Please label the y axes of this figure.

We added “relative expression” to the y-axis labels.

8) Figure 4: Why did the authors separate gene expression for two groups of DCM patients, moderate and severe DCM, but did not perform the same analysis for DNA methylation? Methylation results should be shown for the same two DCM groups and it should be tested whether DNA methylation and gene expression correlate significantly in a regression analysis.

Due to the low amounts of available tissue per patient from the small myocardial biopsies, it was not possible to reliably measure gene methylation and mRNA expression in the same samples. Hence, we are not able to directly correlate gene expression and methylation in the same samples/patients. As noted below, we now performed a promoter activity assay of *LY75* that shows the dependency of CGI methylation with its transcriptional activity (Fig. 6E).

9) Figure 5 & 6: The zebra fish pictures shown in 5A and 6A are identical. Also the functional data for "MO controls" displayed in figures 5D and 6D are identical. This should be clearly stated in both figure legends. In order to find out, whether only one control experiment has been performed, the reviewer finds one paper referenced in the methods section (Meder et al., 2009) which also shows the same control fish (figure 1A) - however with different functional results. The authors should thoroughly address how this could happen and clarify how many control experiments were performed for the present study and which control oligonucleotides were actually used.

The injection experiments of *ly75*-Morpholino (Sequence: 5’-GTGATGAAACGCACACCTCTCCTGA-3’), *adora2a*-Morpholino (Sequence: 5’-CATTGTTCAGCATGGTGAGGTCGCT-3’), and a standard control-Morpholino (5’-CCTCTTACCTCAGTTACAATTTATA-3’) were performed in parallel injection procedures within the same clutches of wild-type zebra fish oocytes (1 cell-stage) to exclude any bias due to the clutch. Hence, the functional investigations of controls were only performed once and compared to the knockdown of the two genes. We now state this more clearly in the materials & methods section.

To also address the comment of this reviewer and reviewer #1, we now additionally performed control experiments with scrambled control-Morpholinos for *ly75* (Sequence: 5’-GTCATGAAAGGCACACGTCTGCTCA-3’) and *adora2a* (Sequence: 5’-CATTCTTCACCATCGTGACGTGGCT-3’), respectively. We added the pictures of these injections in figure 8 and 9 and corrected the unfortunately included image of a standard-control injected zebra fish embryo. We thank the reviewer for identifying this problem.

10) The exact identity of the CpGs, which are displayed in the manuscript should be given in a supplementary table. All array results should be deposited in a public database.

We want to provide other scientists with as much information as possible. Hence, we now included the reference sequences and primers of all tested CpGs in supplemental table 1. Furthermore, the data will be deposited after publication of the manuscript in the Gene Expression Omnibus database (http://www.ncbi.nlm.nih.gov/geo/).

11) Page 31, table 1: The percentage of female and male samples differs between the groups in both cohorts. As DNA methylation is subject to sex-specific imprinting, the groups should be adjusted to rule out gender effects on methylation.

We performed the Exact Fisher Test and found no significant differences of the gender of cases and controls (2-tailed Exact Fisher's test: screening p=1.0; replication p=0.59). However, to follow the line of the reviewer, we also calculated methylation differences after we randomly excluded 1 female from the DCM patients and 2 females from the controls to match the ratio of the screening cohort (replication: females in DCM cases = 33%, controls = 39%). Doing so, we obtain statistical significance for the same CGIs compared to our previous analysis. This is also true when we are matching females and males 1:1 (DCM cases versus controls). We added this information on page 6-7 of the revised manuscript.

12) Suppl. figure 1: The legend should be revised as for most CpGs not even a clear trend for differential methylation is visible. Thus, it would be sufficient to point out HOXB13 as significantly differentially methylated. Please also correct the two different p-values in the legend.

As indicated above, we followed the advice and now present the data on *HOXB13* in figure 4 and not longer in supplemental figure 1. We also clearly pointed out in the legend of supplemental figure 1 that the validation results are not statistically significant.

13) At the end of this study two genes remain which show differential methylation, one up and one down, in DCM while both are expressed at lower levels in severe DCM. It would be important to demonstrate that altered gene expression and DNA methylation are indeed directly linked with each other and that ADORA2A and LY75 are not just by chance occurring on both lists. This is particularly important as both genes show opposite alterations in DNA methylation levels despite reduced mRNA expression.

First, we now have identified the predominantly expressed isoforms of *ADORA2A* in the human heart (ENST00000541988, ENST00000337539). Next, we performed additional MassARRAY experiments for 2 further *ADORA2A* CGIs to identify potential patterns of epigenetic regulation. Interestingly, as shown in figure 4 and on pages 8 in the revised manuscript, we found 1 additional, hypomethylated CGI in *ADORA2A* (CGI-2). Next, we performed an *in silico* TF binding site analysis. Amongst others, we found two strong binding sites for the transcription factor CTCF (CCCTC-binding factor), which is an Insulator-binding protein identified in vertebrates (Bell et al, 1999; Felsenfeld et al, 2004; Gerasimova & Corces, 2001; West et al, 2002).

From the three different CGIs within *ADORA2A*, two showed hypomethylation. In both cases, CTFC binding sites have been identified next to these CGIs. Assuming CTCF to act as an enhancer blocking transcription factor in both cases could explain the down-regulation of mRNA levels together with hypomethylation.

In case of *LY75,* we have now performed a promoter luciferase assay to prove a link between its CGI methylation and mRNA expression. As shown in figure 6E, we see a strongly reduced promoter activity of pCpGL-ly*75* after methylation.

14) As the heart is a complex tissue and its composition changes with disease, the cell type(s) involved in differential methylation and expression of LY75 and ADORA2A should be identified. As the cellular composition of healthy and diseased hearts may differ dramatically and DNA methylation is supposed to be very cell type-specific, it would be important to identify the cell type(s) in the heart which are associated with the observed differences in methylation and expression.

Studies on human tissue provide important insights into the pathophysiology of diseases and allow identification of novel key mechanisms and biomarkers. In contrast to animal models or cell cultures, this avoids the disadvantage of potential inter-species or model disparities. However, tissue samples are often composed of different cell types and as the reviewer notes, in the course of a disease this might change considerably. This is for instance a common problem in most epigenetic cancer studies, were control and tumor samples are considerably different.

In the present study, we have paid special attention to patient selection and the process of tissue sampling. Together, this resulted in high homogeneity of our methylation measurements (e.g. Fig. 1 A). In a study by Grzeskowiak et. al it was demonstrated that differences in gene expression of such myocardial biopsies from controls and idiopathic DCM patients are mainly due to genes expressed in cardiomyocytes and that the contribution of the second most common cell type, fibroblasts, is rather low (Grzeskowiak et al, 2003).

Cell sorting in our liquid nitrogen frozen biopsies was not feasible. Hence, although we cannot rule out that the observed dysregulations are in part due to other cell types than cardiomyocytes, our functional data in zebra fish clearly point towards a major role in cardiomyocytes. The cells of the embryonic zebra fish heart are mostly cardiomyocytes and inactivation of *ADORA2A* or *LY75* leads to dysfunction of these cardiomyocytes and consecutive heart failure. Additionally, we now measured the expression of *LY75* and *ADORA2A* in cardiomyocytes and cardiofibroblasts isolated from neonatal rats and find that both show higher expression in cardiomyocytes.

To better address this important issue raised by the reviewer, we integrated a new paragraph in the discussion section (page 13). Please see also Fig. 7.

2nd Editorial Decision 06 November 2012

Thank you for the submission of your revised manuscript to EMBO Molecular Medicine. We have now received the enclosed reports from the referees that were asked to re-assess it. As you will see, referee #3 indicates that it is suitable for publication. However, referee #1 still raises concerns. His/her main concern is the usage of blood cell DNA rather than cardiac tissue to control for a possible immune- suppressant effect.

We agree that it would be ideal to perform the suggested experiments. However, we acknowledge that obtaining heart samples is only possible by invasive procedures and it is therefore challenging to address this concern. We appreciate that you have analysed global effects in peripheral blood cells, but we understand that it is not entirely clear whether the results can be translated to the heart.

Should you be able to provide the data on cardiac tissue, we would encourage you to include it into the present study. Otherwise, we would strongly encourage you to include a discussion of this issue into the manuscript.

Please include a point-by-point response to the referee's report when you submit your revised manuscript.

Please also address the following editorial point in your revised manuscript:

- The description of all reported data that includes statistical testing must state the name of the statistical test used to generate error bars and P values, the number (n) of independent experiments underlying each data point (not replicate measures of one sample), and the actual P value for each test (not merely 'significant' or 'P < 0.05'). Please see http://onlinelibrary.wiley.com/journal/10.1002/(ISSN)1757-4684/homepage/ForAuthors.html#data2 for more information.

I look forward to reading a new revised version of your manuscript as soon as possible.

Yours sincerely,

Scientific Editor
EMBO Molecular Medicine

***** Reviewers' comments *****

Referee #1 (Comments on Novelty/Model System):

The main issue raised during the first review concerning possible bias due to medication in the control group were not completely addressed. The authors uses blood cell DNA rather than cardiac tissue to control for the possible immuno-suppressant effect. The reviewer details why this might not be adequate in the comment to authors.

Referee #1 (Remarks):

The authors have modified and improved their manuscript taking into account reviewer remarks. However some important points remain unsatisfactory to the reviewer.
A major point raised was the potential bias concerning therapeutic treatment (immunosuppressive) in controls that could affect epigenetic status compared to untreated (or differentially treated) cases. Controls could be explanted hearts without DCM; but these are obviously not easy to obtain. Alternatively, explants or biopsies from other non-DCM cases could have been collected. Instead the authors addressed the question using peripheral blood cells. The mean methylation level of blood cell DNA compared to heart is different (compare fig 5 to fig 3 & 4) suggesting cell specific methylation patterns and potential differential regulation. The authors stated that the methylation level was similar in blood cell DNA before and after transplantation, ruling out any interaction between the four candidates methylation levels and immunosuppressant therapy. It raises some questions:
-If the methylation pattern is similar between blood cells and heart, as implied by the strategy chosen by the authors, why have they not used blood samples to perform the whole experiment rather than "hard-to-obtain" cardiac samples?
-Candidates were ranked on several criteria, one being cardiac expression: what about the expression of the 4 candidates' blood cells compared to cardiac expression (qPCR measurement could be shown). If expression is low or absent in blood cells, what is the relevance of the methylation status regarding expression?
-How and for how long were blood samples drawn before transplantation srored, and what is its possible effect on epigenetic status?
The luciferase reporter assay performed measures complete methylation vs absent methylation of all the CpG contained in the cloned promoter fragment of LY75. With this in vitro setting the difference in luciferase expression is drastic confirming that methylation is an inhibitory epigenetic marker. However, this is not precise enough to demonstrate that the specific and discrete methylation pattern observed in DCM vs controls would have the same effect. Hypermethylation of the 1.6 kb cloned promoter region might very well have different consequences on expression, than discrete methylation of a few CGIs (covering 500nt according to scale indicated fig 3) in the promoter in vivo. This assay is rather non-specific and does not address the question of the effect of the specific differences of CGI methylation on expression in cardiomyocytes.

Referee #3 (Comments on Novelty/Model System):

In the revised version, the authors have substantially revised the manuscript according to the reviewer's suggestions. Thus, the manuscript quality and impact was greatly improved so that I would like to recommend the study for publication.

Referee #3 (Remarks):

I have no further comments - thank you very much for addressing all previous questions.

2nd Revision - authors' response 15 November 2012

Referee #1

Question 1: If the methylation pattern is similar between blood cells and heart, as implied by the strategy chosen by the authors, why have they not used blood samples to perform the whole experiment rather than "hard-to-obtain" cardiac samples? Candidates were ranked on several criteria, one being cardiac expression: what about the expression of the 4 candidates' blood cells compared to cardiac expression (qPCR measurement could be shown). If expression is low or absent in blood cells, what is the relevance of the methylation status regarding expression? How and for how long were blood samples drawn before transplantation srored, and what is its possible effect on epigenetic status?

Answer 1: We have pointed out before that one general problem of epigenetic studies using tissue is the lack of perfect controls. In our case, we relied on patients with regular cardiac function after heart transplantation, in whom we routinely take myocardial biopsies for monitoring. One limitation is the possibility that the immunosuppressives could have an impact on DNA methylation. However, our results excluded a relevant effect of medication on the methylation of our identified CGIs in peripheral blood, suggesting that the medication does not globally change DNA methylation. However, to address the valid concern of the reviewer that this cannot be definitely ruled out for myocardial tissue we have clearly pointed out this limitation in the revised manuscript. For the measurements of DNA methylation in blood, the mean timespan before HTX was 36 months and after HTX 37 months.

Question 2: The luciferase reporter assay performed measures complete methylation vs absent methylation of all the CpG contained in the cloned promoter fragment of LY75. With this in vitro setting the difference in luciferase expression is drastic confirming that methylation is an inhibitory epigenetic marker. However, this is not precise enough to demonstrate that the specific and discrete methylation pattern observed in DCM vs controls would have the same effect. Hypermethylation of the 1.6 kb cloned promoter region might very well have different consequences on expression, than discrete methylation of a few CGIs (covering 500nt according to scale indicated fig 3) in the promoter in vivo. This assay is rather non-specific and does not address the question of the effect of the specific differences of CGI methylation on expression in cardiomyocytes.

Answer 2: We agree with the reviewer that this experiment cannot completely recapitulate the in vivo situation. Unfortunately in in vitro systems, it is not possible to recreate the exact methylation patterns as seen in the primary tissues using the SSSI or other methylases. However, the very well accepted experiment that we performed is further supporting that methylation of the *LY75* promoter is affecting gene expression. To address the concern of the reviewer we have rephrased the statement in the results/discussion sections accordingly.

Referee #3

I have no further comments - thank you very much for addressing all previous questions.

Additional Editorial Correspondence 20 November 2012

Thank you for the submission of your revised manuscript. I am currently in the process of checking it for acceptance, but was unable to find the accession details for the large-scale methylation and gene expression data.

EMBO Molecular Medicine requires that large-scale datasets, sequences, atomic coordinates and computational models should be deposited in one of the relevant public databases and authors should include accession codes in the Materials and Methods section. Please see

http://onlinelibrary.wiley.com/journal/10.1002/(ISSN)1757-4684/homepage/ForAuthors.html#data3 for more information

Could you please provide these by emailing me the relevant sentences, which I will then include in the Materials and Methods section.

I am looking forward to your reply.

Yours sincerely,

Scientific Editor
EMBO Molecular Medicine

Additional Author Correspondence 27 November 2012

Please apologize for the missing information in the materials and methods section of our manuscript.

We have now received the links to the GEO datasets. The updated section in materials and methods reads:

DNA methylation profiling and fine-mapping
For measuring methylation profiles, we used the Infinium HumanMethylation 27 BeadChip assay from Illumina with 1000 ng DNA per sample. The procedure followed the manufacturers standard workflow, starting with the bisulfite treatment of the sample DNA leading to a conversion of unmethylated cytosins to uracil, while 5-methylcytosines remain unchanged. After amplification and fragmentation of the bisulfite-converted DNA, they were hybridized to the Infinium BeadChips. DNA methylation data for DCM patients and controls have been deposited in NCBI's Gene Expression Omnibus (Edgar et al., 2002) and are accessible through GEO Series accession number GSE42510 (http://www.ncbi.nlm.nih.gov/geo/query/acc.cgi?token=lzovlcsmmgomijo&acc=GSE42510). Fig. 1A represents quantile normalized bead color signals (green and red) from 9350 consecutive probes on all further analyzed arrays.

In case of acceptance of our manuscript I will change the GEO status from private into public. If I can be of any additional help please do not hesitate to contact me.
