# Supplementary material for: Alterations in cardiac DNA methylation in human dilated cardiomyopathy
Source: EMBO Mol Med. 2013 Jan 22;5(3):413–29. doi: 10.1002/emmm.201201553 (PMC3598081; doi:10.1002/emmm.201201553)

Table of contents:

Supplemental Table 1: **Primer sequences for MassARRAY assays.**

Supplemental Table 2: **Primer sequences for promoter luciferase assay.**

Supplemental Figure 1: **MassARRAY-based validation of differential methylation in ADORA2A.**

Supplemental Figure 2: **MassARRAY-based validation of genes.**

Supplemental Figure 3: **Protein conservation of LY75**

Supplemental Figure 4: **Regular molecular chamber definition in adora2a-morphants.**

Supplemental Table 1: Primer sequences for MassARRAY assays

Gene	Primer (forward)	Primer (reverse)	Target Genomic Sequence
ADORA2A	TTTTTTAGGTGGGTGTTG<br>GTAGTT	AATTCCCCTAATCCAATA<br>AAATTCC	TCCCTTAGGTGGGTGCTGGTAGTTCCTGGGAGAGTTGACGGGATGGAGCAGGCCA<br>GGGGGGCTGAGAAGTTGGCCTGGCGAGAGAAGGTGGGTGGGACTGGGAGAGCCT<br>GGGCAGGGAGGGGCCTTTCTGGGAGGGAGTGTCATGGGGCTGGCCGAGATCCAG<br>GACCCAGGAGACTGGCCAAGCTGCCACAGGAGGCCTGAGAGGCTGAGCATGGGG<br>GGGCCAACACTGACAGACTTTAACAAGTTTCTGGCTCCCCCAGGAAAAAAACAGAC<br>CACATCTGATCCTTGGCCCTGAGTCCAGAGTGGGAGGCACCGTGACAACAATGCGC<br>AGAGCAGGGAATGCAGGGAGCCATGGATAGTGCTGGGGTGAGCTGGAGTCCTGGC<br>AGGGGCCCAGCTAGGGCAGTACTCATTCTGCAGAGCCTGGGAGAAGGGGCTGCCT<br>CCTTGGGAGGGGTTTGTGTGGGAGTCTCAGCGGGAATTCTATTGGACCAGGGGAA<br>CC
ATP2C1	ATTGGATAAAGGTATAGT<br>TGTTAAAAGAAG	AACAATCACAATCTCAAC<br>AAACAAC	ACTGGACAAAGGTACAGCTGCTAAAAGAAGTGCTCGTTCATTGGCTTCGACAGCTG<br>TACGACTGGTTTCTAATTGCTGTTCCAGCCTCTCCCCAGTTCCATATACCACAAACG<br>AATGAAAAAGATTAGCTCTCCCTGGAGCTCTCTGAGGCTGAACCTTGCTCTCTCATT<br>ACACATGGTAACTAAGCACACCCCTTAAAGAATTTTGGGAAGTCGCCCCGCGATTAC<br>CGGCTCCCCCAAGAACCAAGCAGTCAGTGCAGGGTTTTGCAAGTGAGAGTGCGAG<br>ACTCGGGAGGCTGCTCACGGTGCTCCAGTCCCTGGCAGGAGCATGGACACCTCGC<br>CCCTTCCTTCCGTGGGCCTCAACGTTGCCTGCTGAGATTGTGACTGCC
CCDC59	TTTTTAGTGGAAGTAGAG<br>TGGGAAG	TTAAAACAACCTCCTTAC<br>ACAATCC	CCCCTAGTGGAAGCAGAGTGGGAAGCAGGTGGGTGGTGGGGTAGGCGGGGCGGG<br>AAGGGAGGCGGAGGAGAAGGTCCCGGCAGACGAAGCGAGCCCCCTGGTGGAAGC<br>CAGCAGAACCAGGTGCGTGGCGGCGGAGGCGGGGCGGCCCGCTGGGAAACCGA<br>GGAGGGGTCGGATTAGTTGAGGGGTGGGGTGGGGATGTTCTTTGGAATCAGGATC<br>ACTCTTTCTTTGGGTGCTTGTGTATGTCCACAATTTAAAGAAAGCAGGAAACAGGGC<br>AGGGGAGTTCCTTCATCTAGTAATTCCATAATAATTGACGCTAGGGAAACTAGGGGA<br>GCAGTGCTCTCACAGGTTTTATAAATGCCAGCATGTGGTGGAAGATTACTCAACAGT<br>TAATAAAGATAATTTCAAGTGGTGGTAATGAAGTAGAAAATAAAAACAGTGTAATGTG<br>GTAGGGATTGTGTAAGGAGGCTGCCTCAA
CLDN4	GTTTATTTATAGGTTTTTT<br>TAGATGGTTTG	CATCAAAACTAACTTTAT<br>CTCCTAACTCA	GTTCATCCATAGGCCCTCCCAGATGGTCTGCGAGGTGACAATGTTGCTGCCGATGA<br>AGGCCGTCACGCGCCACATGGGCAGCGCGCAGCACAGCATGACGGCCAGCCAGC<br>CCAGGACGGCCAGCGCGATGCCCATTACCTGTAGCCCCATGGAGGCCATTGTTCA<br>GCGTCCACGGGAGTTGAGGACCTGGAAGGCTGAGGGGATCCGGCTGTGGGGGCC<br>GTAGGATTCCAAGCGCTGGGGACGGACTTAACGTTCGCAGAGTGCACCTTTGCACC<br>GTGAGTCAGGAGATAAAGCCAGTCCTGATGC
ERBB3	GGAGATTTTTTAGTAGAG<br>AATAGGTTTTTT	TACTACCCAAAACCCTAC<br>TAATCCC	GGAGATTCTCCAGTAGAGAACAGGCCCTCTAGGTTGCATATCAATAGGGAGCATGT<br>TTAAGGAATGTTAGCCGGTAGTCTTTGCTAGGTGTGAGGGGTGAAATTTTTCTTTAT<br>CAAGGCTCAACTGTTTTCGAAGTCTTCAGGCTTGAAGTTCTGGAGAAAACAACTAGG<br>CTCTCCGGGCGAGATCCCGAATACCAGTTTAAGGGATTTGAAATGCAAGGCCGTCT<br>GGGACTCCACTGCCACGGATGGGCACCAGGCGGCGCCGGTCGGATCCGTCCCGG<br>GACTAGCAGGGCTTTGGGCAGCA
FDX1	TTTGTTTTATAGTGGGTA<br>GAATTTATTT	AACCTATAAAAACATTAA<br>CAAACAAAAC	TTTGTTTTACAGTGGGCAGAATTCACTTTGCTCTAACTTGACTCCAAGATGACACAAT<br>TTCCCAGTTTTTTTTTTAACGGCTAGGGGATGGGGTATGCGGAACAGAACACAGT<br>GGTCTAGCTAACCGATTTCCGCTAGACGTCCAGCTATCTTTCAAAATATTTTGTTTCT<br>GCACGGCAACTTCAGCCGCTAAAAAAGCATCCAGCTTACAACGGAACCTGGAGGGT<br>TGGTAAAGGCCCCCTGCGCTGGCCCCGCCCCATGGGACCGGGCGGCGTGGGCGT<br>GAGAGGCGGGGCGGGGCCGCGCTCTGCTTGCCAATGTCTTTATAGGTC
FLJ20701	GATTTTAAATTTTAGTGA<br>GGGGTGAA	TCCAAAACAAAAAATAAA<br>CTCCAAA	GACTCTAAATCCTAGTGAGGGGTGAATGGGGAGGCTAGAACAGCCGGCCCTTCCCT<br>GCAGGAAGCCCCCATTGGGTTGGGGTCTGGGAAAAGGGACAGACTGGCTGAGGG<br>GGGAAGGGCCTGGAGCCGGGGGTGGTGGGGGCAGCGGGGATGTCGGGGGTGGC<br>GGGGGTGGGGGAAATTCCTAAAATAAGGAAACCGTAGCCCCGGGGGTCCCGGGTT<br>TCCATATAACAGGCATCTCTGAAGTGCCTGTGCTGTGCCTGGCGCTGTGCTAGGGA<br>GACACTTCCATTCATTCTTCCCACTCCAGTTAACTGAGCATCTATTAGGCTCCAGCC<br>CCTATGCTGGGTTCTGAGGATGCAGCGACGCAGAATAGAAGACAAGAGAATGTTCC<br>TTCTCTCTGGAGCTTACCTTCTGCTTTGGA
GFI1	TTTATTTTAATGAGTAAA<br>GGGTGTAGGT	CCTAAAATCATACCCAAA<br>CACTAAAT	TCCACTCCAATGAGCAAAGGGTGTAGGCTTTGAGTATACACAGCAGGCCACACACT<br>ATGGGTCATGGCATACAGTCTAGCGTTGGAGGTTGCTGCATTTGTTCATGTATCTAT<br>GAGTTAGGTTTTAAGTTTTTTCCCCCTGTTCTATTAATATAATTAATTAGTTAATTAAC<br>CCCACTCATTCATTTACTCAGTCCACAGCCTTTTGTTAAGAGCCCGCCTAGGACATA<br>AAGATGAAGATCCAGTGCCTACCCTCATGAAACATAGAATCCGAGCGAAGAAGGCT<br>TACTTTGCAATAGTCATCCAAGTGGTGCTTTTATGGCGCTTTACTGCTCCCGCCAAC<br>ACCCAGTGCCTGGGTATGACCTCAGG
GSTM5	GGAGGGGGTTTATTGAT<br>TTTAGTTT	TAACTTTCTCTACACCAA<br>ACCAACC	GGAGGGGGCTTATTGATTCCAGCCCCGGGCGCTCGCTCGGGGGCCTACAGAATGG<br>CGTGTTTCGGGGTTGTGGCGGGCCGAGGGGCGGGGTCGCAGCAAGGCCCCGCCT<br>GTCCCCTCCTGGGCCTCTCAAAGTCTGAGCCCCGCTCCGCTGATGCCTGTCTGCAG<br>AATCCGCACCAACCAGCACCATGCCCATGACTCTGGGGTACTGGGACATCCGTGG<br>GGTAAGCGAGGGTCCTCTGGTGGGTGGGACAGGGGGCGGAGGCGGGGATGTGTG<br>GAGTAGCTGCAGGACTGGCTCTAGGGACCGTTCCTCTTCAGGGCTGCCCGCCTCA<br>GAAGGGCCTGTGCATGACGCTGTGTGTGTGTGTTTGGGGGTGGGGGCGGGTAGAGGA<br>GGCGACGGGTACGTGCAGTATAGACTAGGGGCTGGCCTGGTGCAGAGAAAGTCAC
HOXB13	GGTAGTTTTTGGTTTTGG<br>GTTTT	ACTATCCCTAAATCTCAT<br>CTCTCCC	GGCAGCCCCTGGCTTTGGGTCCTGGGTACTAGCACCCCAGTTCATTCCCGGTACCC<br>TTCCTGTCCCAGATGTCCTGGAATGAGCTGCTCCGGGCCCCAGGGAGGTGCCCTG<br>GACGCCCCTTACCCAGGGACTCGCTACTGCCCAGAATGCTCTGGAGGCCCTGATTC<br>GGGTGAGGCTGAGGGAAGAGGCGGGAGACACGAACGTAGACGTAGAAGACATGC<br>GATCTGGATGGAACCCAGGACACTCCACTTCCGGACCTGAAGGGGAAATGGAGCC<br>TCTAGAGTGGCCTGTGGAAGGGTCTGGATTTCCCTTTAAGTAAAGACATACGGAGG<br>GACGGAGACCCACAGGACTTGAGGGAGGGACGGGGAGAGGGAGAGATGAGACTC<br>AGGGACAGC
ID4	AATGGAGTGTTTTTTTTA<br>TTGGTT	AATATCCTAATCACTCCC<br>TTC	AATGGAGTGCCCTTCCCATTGGCCCGAGCGTCATTCCCCGAGGTGGCACTGCCCG<br>CCTGATTGGCTGGCCACTCCAGACCCCCCGCCCACTCCTCCACTCGGGTAGCCGG<br>ACTCCCCGCCCCCCAGCACCGCCCGGAGCCCCCGCCCTCGCCTCTCCCTCCGCGC<br>CCCCGCCCGCGCGCCCAGCGGGCTCCGCTCGGCTCGCGCTGCGACCCGGCCCGC<br>GCGCTGGTCCCGCCCCCGGGGCGCACGGCTCTATAA

LY75	GTATTTTTGTTTAGGTTT<br>TTGGGG	AAAAATCATAAATCCCTT<br>TCCAATC	GCACCTCTGTCTAGGCCTCTGGGGGCGCCCCGGCCCCGCCCCCGCCGCCCTCGG<br>CCAATCAGACGTGCGTCTCCTCGGCCCCGGGGCGGAGCGGGCCAGGTGTGGGAA<br>ATGAACAGGGCTGGGCGCTAGATACCTGCGTGGGGTAGGACCCGCGAGGAAGAG<br>GTACGTGCGGATCGGTGGGAGAGCCAGGCACCAGACAGGCTCCTGCACTGGAGG<br>GTTCGGTCCCCGCCTCTTCATCAGCCAAGCTGGGGAGATGCGGCCCTTACTGGGA<br>CTTGGCACCGCCCTGGTGGGTGGGTTCTATCAGTTTAGAACCTTGGCCTCTGCCTG<br>GCGCACTGTGGTCAGGGACGACTTCTCCATTCCAGCCTGGACTGGAAAGGGACCC<br>ATGATCTCT
NAT1	TTTTTTTAGGAAGTAAAA<br>GGAATGTT	TCAAACTACCCTAATCTC<br>ACTATCCC	CCTCCTTAGGAAGTAAAAGGAATGTTAGTGTGGGATTTTGCATTGGAGGCTAGTGCT<br>GACCCCACTGCAGTGGAACACAGCACTGGGCAGAGCCTCCAAGGCCCTTGATTCTA<br>GGCTGGAGGCCACAGAGGGAGCATTTAGACCCCCGCCCCGGGTCAGAGGGAAATC<br>TGCTACCTCAGCAGGAGGAATCCAAGTTCCAGTCCACTTCACAACCACCTAACTAAA<br>GTGGCCTTCCGCCAGACCCTAAAACAAAGATTTGCATTCAGGTAGATTATTTGGAAG<br>ATGATTTAAGGACGTACCAGTGCAGGAGTTGTCGCGGGACAGTGAGACCAGGGCA<br>GTTTGAC
PPARGC1A	GATTTTTTTAAAAGTTTTT<br>GTTTGG	ACAAATACCTTCAATTCA<br>CTCTCAA	GATTCTTCTAAAAGCTCCTGCCTGGACTACTTTCCTGGCTCCTCTCTTTGCCCAAGC<br>CCATCCCCCCTTACAGGAATAATAGCATCTGAGGGAAGCGTCAGTTGTGGCTGCAG<br>CGCCGAGCCCTGCCCCAGCTCACCTCGATGTCACTCCATACAGACTCAGAGTCCTG<br>GTTGCACATGTCCCACGCCATCCAGCTCCTGAATGACGCCAGTCAAGCTTTTTCAAC<br>TCCAATCCACAGTGACACAGAGCACACACTCATGCAGGCAACCAGCCCCTTACTGA<br>GAGTGAACTGAAGGCACCTGT
SLC9A6	GTTTTTGATTGGGTAGG<br>GGT	AACTTACCATAAATCATA<br>ACCAAACC	GTCTTTGACTGGGCAGGGGCTTCGGACGGCGGCGGCGGAGAGGCTAGAGCCATG<br>GACGAGGAGATCGTGTCCGAGAAGCAAGCCGAGGAGAGCCACCGGCAGGACAGC<br>GCCAACCTGCTCATCTTCATCCTGCTGCTCACCCTCACCATTCTCACAATCTGGCTC<br>TTCAAGCACCGCCGGGCCCGCTTCCTGCACGAAACCGGCCTGGCTATGATTTATGG<br>CAAGTTC
SULF2	GAGTTTTGTGAAAGTAGA<br>TAAAAGAAAAT	TAACACACAAACCAAAAA<br>ACATC	GAGTCCTGTGAAAGCAGATAAAAGAAAACATTTATTAACGTGTCATTACGAGGGGAG<br>CGCCCGGCCGGGGCTGTCGCACTCCCCGCGGAACATTTGGCTCCCTCCAGCTCCG<br>AGAGAGGAGAAGAAGAAAGCGGAAAAGAGGCAGATTCACGTCGTTTCCAGCCAAGT<br>GGACCTGATCGATGGCCCTCCTGAATTTATCACGATATTTGATTTATTAGCGATGCC<br>CCCTGGTTTGTGTGTTAC
TDG	TGGTTGGTAGTATTTAGA<br>TAGTGGTTG	ATATACACAACACCCCAA<br>AAACAA	TGGCTGGCAGTACCCAGACAGTGGCTGGACTCAAGCTCCTCCTCCAGGCTTCTACC<br>GTCCCCCACGGACCCCCTTTCTGTGCTGCACTTCCGCCTCCTCGGAGCCAAATCCC<br>ACGCGTACTGGCCGTCACGTGATGTAGGACAGCCCAATCACGCTCACCCCCTTCTC<br>GTTGGCGCCCCAGCTCCGCCGCTTGGCCTTTCGGGAAGGGTGGAGGACCAGAAGA<br>CGGGGACTAGGAGCGCTCTGCCGACGTCAGCTTTTGTCTCCAGTATTTTCGGGGGC<br>TGGTGGACGCGTGGGCGATAGGGTGCTGTCCTTGGGGTGCTGTGTATAT
TFF1	AGGTTGTTAGAGTTGGTT<br>GTGGTT	AACTTTCTAAATCTCAAA<br>TCCCTCAA	AGGCTGCCAGAGCTGGCTGTGGCCCCACAGAGCAGGAAGAAGCACGCCTTACCTG<br>TCTGGGCCTCGGCCAGGGTGCCGAGGGCCAGCATGGACACCAGGACCAGGGCGC<br>AGATCACCTTGTTCTCCATGGTGGCCATTGCCTCCTCTCTGCTCCAAAGGCGACCC<br>CGAGTCAGGGATGAGAGGCCGCCCGAGCCCCGGATTTTATAGGGCAGGCTCTGTT<br>TGCTTAAAGAGCGTTAGATAACATTTGCCTAAGGAGGCCCGGGGATCCTCTGAGAC<br>AATAATCTCCACTGATTTTTATCAAAGGTGTTTCCTAGACATGGTCAAGCTACATGGA<br>AGGATTTGCTGATAGACAGAGACGACATGTGGTGAGGTCATCTTGGCTGAGGGATC<br>TGAGATTCAGAAAGTC
TKT	GGGAAGGTTATTATTATT<br>GTTGTTTT	AAATCCCAAAATTCTACA<br>CCCA	GGGAAGGCCACCATTACTGCTGTTCTACAGAACGCGGCCCTCTTAACCCTCTCCAC<br>CACCTGGCTGTGTGGGCGCCGCCGGGAGTGTTCCCCCTTCCAGATGGGGAAACGG<br>AGGCTCCGAGTTAGTCAGCTACCCAAAGTTACACACAGGGAGATCTGATTTGAGCC<br>CCAGCAGTCCGACTCTAGAGACCCTGTTTTTAATTCCTCTTGCAGGAGACTCGGGG<br>GCCCGGCCTTCTCCCCACCTCAGACTACGCTCCCCTACCCCTGTCTCCTGCCACCC<br>CTGGGTTGGGTCCGAGTTTGGAGCAGGGGGCTGCTGAGACGCCAGGCAGCGGCG<br>GGGCTGATTGGGGGAAGAGGGGAGGAGGGCGGTGGCACGTGGGTGCAGAACCCT<br>GGGATCC
XRCC5	GTTTTTTGTTAGGTTTGA<br>AAGGGG	AAAAAAAATCTAACTCCA<br>AAACTCTAA	GTTTCCTGCTAGGCCTGAAAGGGGCGGGGAAACCGTGCCCCGGAACTCTGAGCAT<br>GCGCAGATTCCCGCCGAGCTTGCGCAGATTCCCGCCGAGCTCGCGCAGATTCCGG<br>CCGAGCATGCGCAGATTCTCTCCGAGCATGCGCAGATTCCCGCCGAGCATGCGCA<br>GATTCCCGCCGAGCATGCGCACATTCTCTCCATTCCGCCGTAGTCGGGAGCGTGTG<br>GTGTTTGAGCCGCCTGAAACGCTAGTACGACAGATTTACTTAAAGATTCACTTAATC<br>ACTTAAAGATTCACGTCCCTTTTCTCTCATTGAGAATAACACCTAAGGCATTTTGTGT<br>CAGAGCTCTGGAGTTAGATTTCTTTT

Supplemental Table 2: Primer sequences for promoter luciferase assay

Gene	Primer (forward)	Primer (reverse)	Target Genomic Sequence
LY75	TGGCACTACCCTGAACA<br>TCA	CATGAAATTTCTGGTTCT<br>GCAT	TGGCACTACCCTGAACATCAATGAAGGCTGTCTGTTTCTGGTCCTGCTTCCGCAGTG<br>AGAGGCAGCAGGTCCCCAAGCAGGCGACCTGCCCTTCACCGTGAGGCTGTCAGCC<br>TTGGGGGAGCGGGGTCATTTGGAGCTGATAAACCAATGAGAAGAAAGGTTTGTTGC<br>TCTAGGCGGTGGGTGAGGGCATCATAGCTGACTCTTGGTCTTGGTCACTTTCGGAG<br>GAGATGGTTTATTTAACCTGACTTCCTTCCTGATGCGCACCGTAGGCGCAGTGAAAT<br>CCGGGAATCGTGGGGAATCCTTGGCGCTGTGGGTGGAGGCTCCTCTTGGCCCTGT<br>GGCCAAGGTGACCAAGGGCCGAAGGAAAAGCGAGAACGGGAGGGACGGGACGCA<br>AGAGGGCAGATGGGGAACCCCATACTCCAGCAACATTATATAAGAGAGGCGACGAT<br>GGAGCAGCGCACCCGGCCAAAAAAGCCTCCGTGCGCCTACTCTACGGTGCACCGC<br>GTCCCCTCTGCACCAGAAGGGCCCTGTCCTCCCACATCCACCGCGCCCTCCTCCG<br>GGCCCCCGAGGGCACTGGGGCGCTTCCTCTGCCAGACCTCCCCTGCGACTCACTC<br>TTCCGGCTCCAGAGCCCCCCCGCCCCAACAGCAAAGCAGCCGTGACCTGCCCCAG<br>GGGCGCAGCCCTGCCCCAGGCTGGAAGGCAGCAGAGCTGTGGCGTCGAGGCACC<br>CAGCGGACTGCGGGGCTGGCGTGCCCGCGGTTACCTGCGCGGCCAGAGGGCTCC<br>GCGAGATCGAAGAACCAGAAGAGCAGCATGAGGAGCCCCGCCGGGCGGCGAGGG<br>GTCGCCCAGCCTGTCCTCATCCTGAGCTGGCGCAAGCCTTCCGGCCGGGTCCTCG<br>GGCGCACGCGGCTCCCGCCCCGCCTGCTGAGCGCGGCCTGCCCCGCCCGCACCT<br>CTGTCTAGGCCTCTGGGGGCGCCCCGGCCCCGCCCCCGCCGCCCTCGGCCAATC<br>AGACGTGCGTCTCCTCGGCCCCGGGGCGGAGCGGGCCAGGTGTGGGAAATGAAC<br>AGGGCTGGGCGCTAGATACCTGCGTGGGGTAGGACCCGCGAGGAAGAGGTACGT<br>GCGGATCGGTGGGAGAGCCAGGCACCAGACAGGCTCCTGCACTGGAGGGTTCGG<br>TCCCCGCCTCTTCATCAGCCAAGCTGGGGAGATGCGGCCCTTACTGGGACTTGGCA<br>CCGCCCTGGTGGGTGGGTTCTATCAGTTTAGAACCTTGGCCTCTGCCTGGCGCACT<br>GTGGTCAGGGACGACTTCTCCATTCCAGCCTGGACTGGAAAGGGACCCATGATCTC<br>TTCTACCCCGGAGGAGGAAGTGAGCACCTGCCCTGTGGGTGGCTGCGGCCAAGCC<br>TAAGAATTCAGTCGTCCTTGGCAACGTCTTGGGTATTTTGACAGTGCAAACAAAGGT<br>GGAATAATGGTACACTGCAGTTCTACCCATAGTTGTTAAAGAATTAAAAGCAAGAATT<br>ACTAGAATGACCAAACGACACTTCCAAGGATGACTTATGCTTTATAAAAAGTTGACCT<br>TTGCGAGTAAGCTCTTTGCTTAATAATTTAATGATAATAATAATTAGCTGGTAGAAAT<br>GTAGAAGTCTGCATGCAGAACCAGAAATTTCATG

Supplemental Figure 1: MassARRAY-based validation of differential methylation in *ADORA2A*. Graph representing individual CpG methylation in *ADORA2A* measured by MassARRAY in DCM patients and controls.

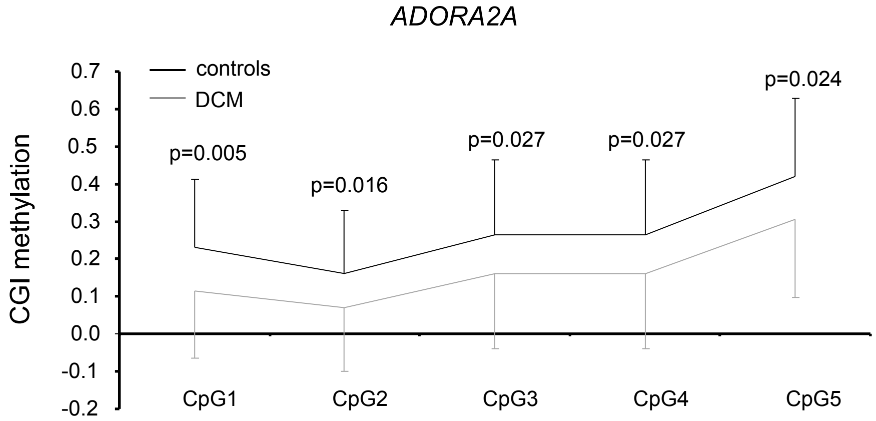

Supplemental Figure 2: MassARRAY-based validation of genes. Graphs representing the mean CGI methylation of targets included in the replication stage. DCM patients show a statistically not significant reduction in methylation level in *CLDN4, FDX1, HOXB13, ID4, NAT1, PPARGC1A, SULF2, TFF1, TKT* and a statistically not significant increase of methylation in *ATP2C, CCDC59, GFI1, GSTM5m, SLC9A6*, and *TDG*.

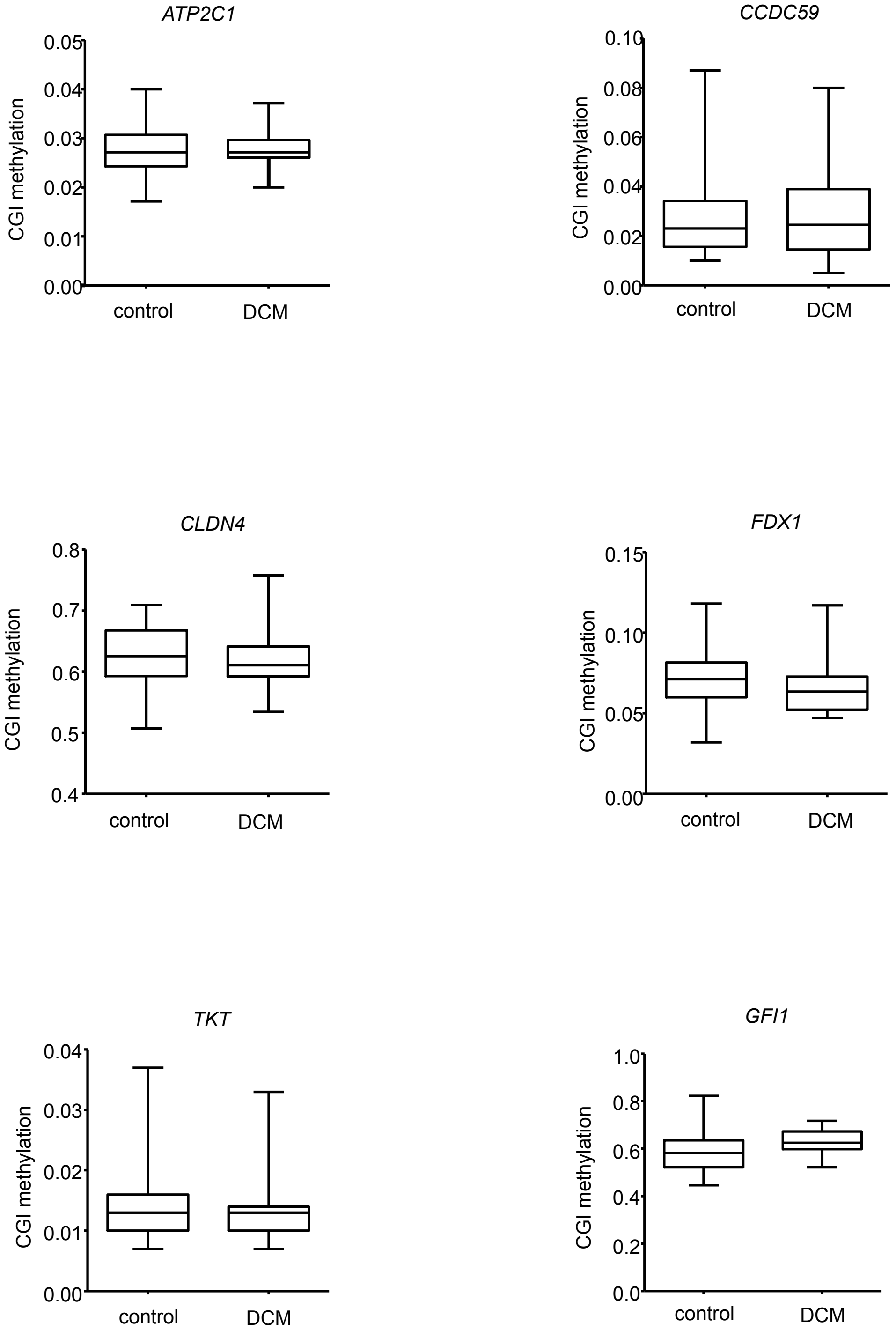

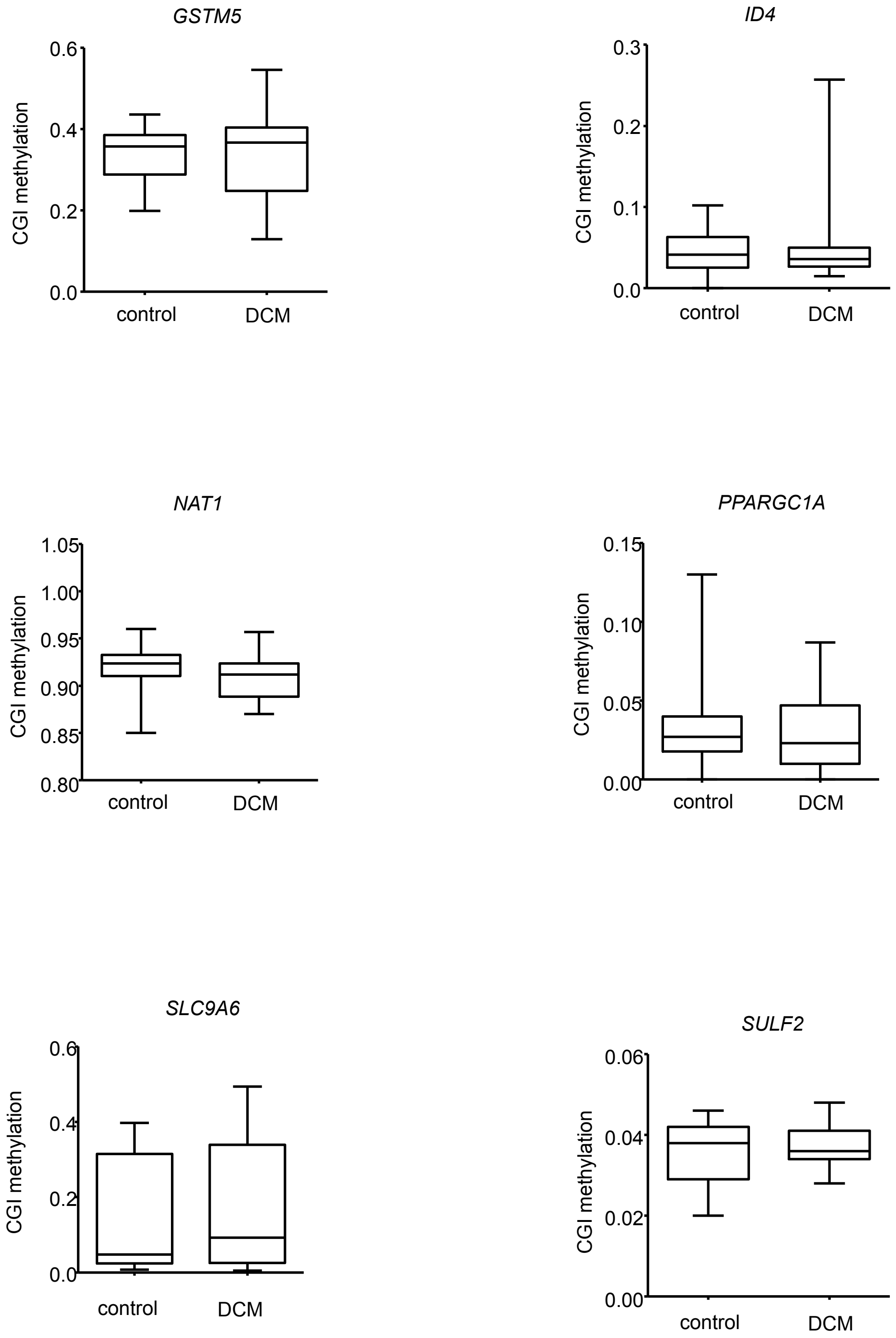

GSTM5
CGI methylation
0.0
0.2
0.4
0.6
control
DCM
ID4
CGI methylation
0.0
0.1
0.2
0.3
control
DCM
NAT1
CGI methylation
0.80
0.85
0.90
0.95
1.00
1.05
control
DCM
PPARGC1A
CGI methylation
0.00
0.05
0.10
0.15
control
DCM
SLC9A6
CGI methylation
0.0
0.2
0.4
0.6
control
DCM
SULF2
CGI methylation
0.00
0.02
0.04
0.06
control
DCM

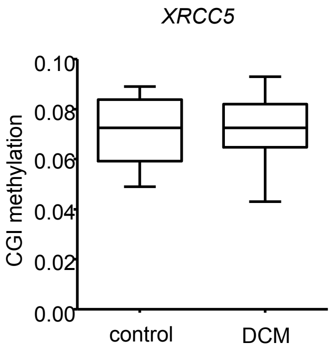
XRCC5
CGI methylation
0.10
0.08
0.06
0.04
0.02
0.00
control
DCM

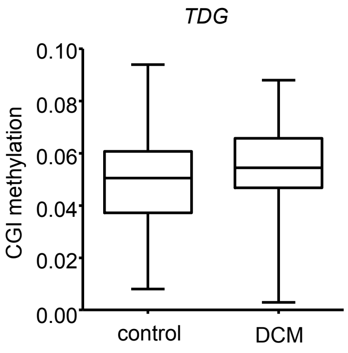
TDG
CGI methylation
0.10
0.08
0.06
0.04
0.02
0.00
control
DCM

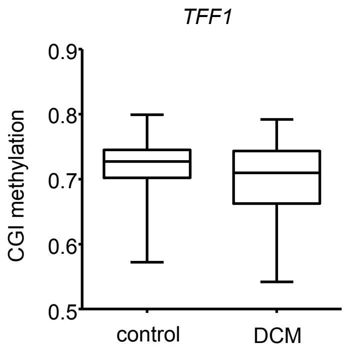
TFF1
CGI methylation
0.9
0.8
0.7
0.6
0.5
control
DCM

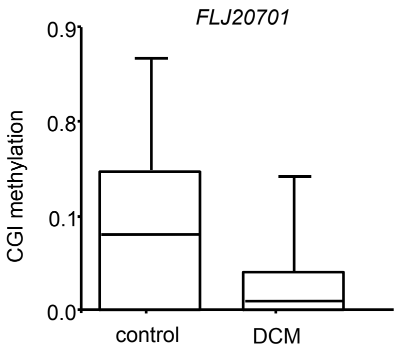
FLJ20701
CGI methylation
0.9
0.8
0.1
0.0
control
DCM

Supplemental Figure 3: Protein conservation of LY75. Amino acid sequence alignments of human (h), mouse (m) and zebrafish (z) LY75 demonstrating its cross-species homology. Black boxes indicate amino acid identity, gray boxes indicate amino acids with similar chemical properties.

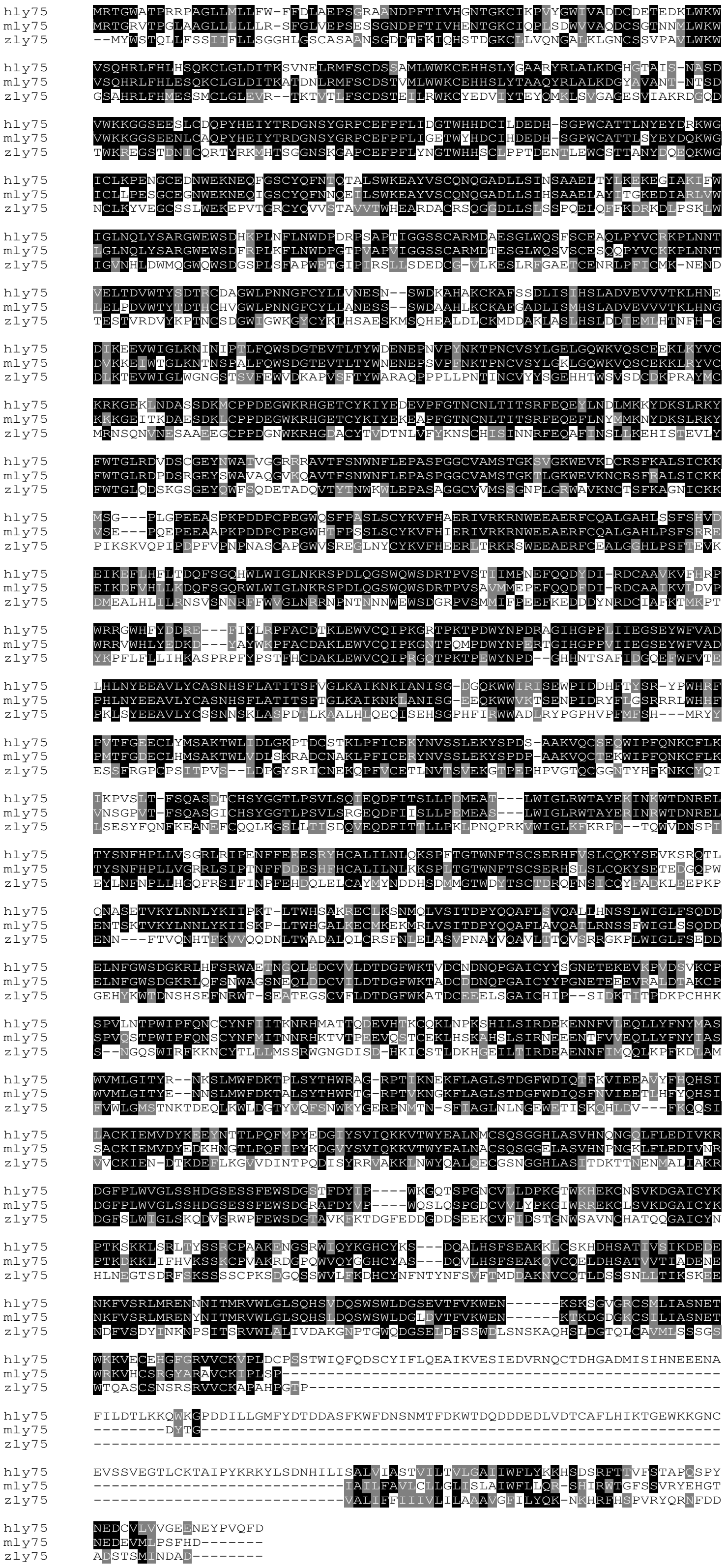

Supplemental Figure 4: Regular molecular chamber definition in *adora2a*-morphants. Molecular chamber definition is not impaired in *adora2a*-morphants compared to the control-injected zebrafish as demonstrated by regular *amhc* and *vmhc* expression.

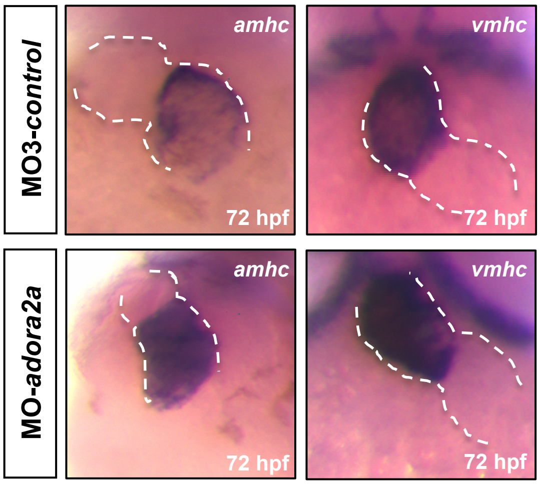

Supplement: Supplementary file 4 [file emmm0005-0413-SD4.pdf]
